# Schizasterid Heart Urchins Host Microorganisms in a Digestive Symbiosis of Mesozoic Origin

**DOI:** 10.3389/fmicb.2020.01697

**Published:** 2020-07-22

**Authors:** Alexander Ziegler, Ariel M. Gilligan, Jesse G. Dillon, Bruno Pernet

**Affiliations:** ^1^Institut für Evolutionsbiologie und Ökologie, Rheinische Friedrich-Wilhelms-Universität, Bonn, Germany; ^2^Department of Biological Sciences, California State University, Long Beach, CA, United States

**Keywords:** Echinoidea, microbiome, digestive tract, intestinal caecum, spirochete, *Brisaster*

## Abstract

Because of their lifestyles, abundance, and feeding habits, infaunal marine deposit feeders have a significant impact on the ocean floor. As these animals also ingest microorganisms associated with their sediment and seawater diet, their digestive tract usually contains a diverse array of bacteria. However, while most of these microorganisms are transients, some may become part of a resident gut microbiome, in particular when sheltered from the main flow of digesta in specialized gut compartments. Here, we provide an in-depth analysis of the structure and contents of the intestinal caecum (IC), a hindgut diverticulum found exclusively in schizasterid heart urchins (Echinoidea: Spatangoida: Schizasteridae). Based on specimens of *Brisaster townsendi*, in addition to various other schizasterid taxa, our structural characterization of the IC shows that the organ is a highly specialized gut compartment with unique structural properties. Next generation sequencing shows that the IC contains a microbial population composed predominantly of Bacteroidales, Desulfobacterales, and Spirochaetales. The microbiome of this gut compartment is significantly different in composition and lower in diversity than the microbial population in the sediment-filled main digestive tract. Inferences on the function and evolution of the IC and its microbiome suggest that this symbiosis plays a distinct role in host nutrition and that it evolved at least 66 million years ago during the final phase of the Mesozoic.

## Introduction

All animals are immersed in a microbe-filled world and interact with microorganisms across body surfaces exposed to the environment such as the integumentary, respiratory, urogenital, or digestive systems (McFall-Ngai, 2015). In many cases, microbial communities (or microbiomes) distinct from environmental bacterial communities reside on these surfaces or inside specific body compartments (Shade and Handelsman, 2012; Dubé et al., 2019; Zhang et al., 2019). The diverse metabolic activities of these microbiomes are often of critical physiological importance to the host, as is perhaps best exemplified by the many functions of the gut microbiome: protection against colonization by pathogens, development of the host’s immune system, breaking down toxic components of the diet, digestion of refractory components, and provision of small organic molecules valuable to the host organism (Dubilier et al., 2008; Cavanaugh et al., 2013; Sommer and Bäckhed, 2013). In fact, gut microbiomes are of such importance to their hosts that they have significantly influenced the evolution of metazoan digestive physiology as well as gut development and morphology (McFall-Ngai et al., 2013; Graf, 2016; Moran et al., 2019).

However, although digestive symbioses are known to occur in a wide range of metazoans, not all animals appear to establish a gut microbiome: in some species, the only microorganisms routinely found in the digestive tract are ingested with the food, and are therefore only transiently present (De Ridder and Foret, 2001; Hammer et al., 2019). One guild of animals for which this may generally be the case are marine deposit feeders, which often occur at high densities over large areas of the ocean floor (Jumars, 1993), and whose feeding and burrowing activities have significant biogeochemical consequences (Lopez and Levinton, 1987; Rosenberg, 2001). Most marine deposit feeders possess simple, tubular guts through which ingested material passes in a continuous, rapid flow (Penry and Jumars, 1987, 1990; Jumars, 2000), potentially hindering the establishment of a gut microbiome. However, even in such a scenario, theoretical considerations suggest that the potential for microbial activity might be particularly high in the hindgut, and that marine deposit feeders should be able to take advantage of this localized activity by retaining stable bacterial communities inside evaginations or pouches (Plante et al., 1989). Such hindgut diverticula have been observed in several marine deposit feeders, but evidence of microbial activity in these organs is sparse (Plante et al., 1990; Harris, 1993; Jumars, 1993).

In this study, we provide a detailed description of the structure and contents of such a hindgut compartment, the intestinal caecum (IC). This particular organ is found in some members of a widespread and abundant group of marine deposit feeders, the schizasterid heart urchins (Echinoidea: Spatangoida: Schizasteridae). Spatangoids are ubiquitous infauna on the continental shelf (Nichols, 1959; Moffitt et al., 2015), and as active burrowers play an important role in marine benthic ecosystem functioning through bioturbation (Bromley and Asgaard, 1975; Hollertz and Duchêne, 2001; Widdicombe et al., 2004; Lohrer et al., 2005; De Gibert and Goldring, 2008). Their activities result in transport of the organic-rich surface layer into deeper sediment layers and thus enhance benthic production both in shallow and deep sea habitats, while additionally increasing the diffusion of oxygen and uptake of carbon by the sediment community (Pequignat, 1970; Osinga et al., 1997; Hollertz, 2002; Vopel et al., 2007; Boon and Duineveld, 2012). Schizasterids, for example, possess a range of morphological features that permit burrowing in silty or muddy sediments, habitats that are frequently characterized by extreme hypoxia (Mortensen, 1951; Chesher, 1963; Buchanan et al., 1980; Kanazawa, 1992; Schinner, 1993; Bromley et al., 1995; Thompson and Riddle, 2005; Sato et al., 2017). While the main digestive tract of spatangoids is packed with inorganic and organic particles taken up from the ocean floor, structural derivatives of the gut have been shown to be devoid of sediment: these functionally specialized organs include the primary and secondary siphon (De Ridder and Jangoux, 1982; Thorsen, 1998), the gastric and recto-intestinal caecum (Ziegler et al., 2010; Rolet et al., 2012; De Ridder and Saucède, 2020) as well as the IC discussed here. Given its position and the fact that the IC has been shown to contain a flocculent mass instead of sediment (Ziegler, 2014), we predict that this organ could be the site of a complex, yet undescribed digestive symbiosis. In a distantly related group of heart urchins, a similar structure was previously shown to contain nodules composed of a diverse array of microbial taxa that assist in digesting bulky inorganic or organic components forming part of the sediment diet (De Ridder et al., 1985; da Silva et al., 2006). This raises the question whether similar mechanisms could have independently evolved in schizasterids as well.

To better characterize the IC and its contents, we examined the well-studied and relatively easily obtainable schizasterid *Brisaster townsendi* using a diverse set of imaging techniques, including magnetic resonance imaging (MRI), micro-computed tomography (μCT), light microscopy (LM), scanning electron microscopy (SEM), and transmission electron microscopy (TEM), complemented by next generation sequencing (NGS). In addition, we obtained structural and genomic data on the IC from a representative of the schizasterid genus *Abatus*. This taxon has recently emerged as a model for ecological, biogeographical, and evolutionary research on benthic invertebrates of the Southern Ocean (Linse et al., 2008; Féral and Poulin, 2011; Michel et al., 2016; Fabri-Ruiz et al., 2017; Guillaumot et al., 2017). Finally, we compared our structural and volumetric observations of the IC in *Brisaster* and *Abatus* with other schizasterid taxa to establish inferences on the timing of origin and evolution of a potentially highly specialized organ found in an abundant group of marine deposit feeders.

## Materials and Methods

### Specimens

Living individuals of *Brisaster townsendi* were obtained in May 2018 by otter trawl off the coast of Southern California on the San Pedro Escarpment (33°40.802 N, 118°19.764 W, 266 m depth to 33°41.016 N, 118°19.999 W, 275 m depth). This upper continental shelf soft sediment site lies within an area systematically monitored over the past decades with regard to various environmental and ecological parameters (Thompson et al., 1987; Thompson et al., 1993; Allen et al., 1999; Stull et al., 2001; Sato et al., 2017; Munakata and Markle, 2018). During the ca. 1 km long transect on a southwest-facing slope parallel to the coastline, several hundred individuals of *B. townsendi* were captured, in addition to numerous specimens of *Strongylocentrotus fragilis* and other infaunal and epibenthic animals. The *B. townsendi* sample was compared with eleven specimens of the closely-related schizasterid *Abatus cordatus* collected by SCUBA in the Golfe du Morbihan, Kerguelen Islands in November 2015 off Port-aux-Français (49°21.15 S, 70°13.083 E, 6 m depth) and in December 2015 off Île Suhm (49°30.90 S, 70°09.14 E, 10–15 m depth). Additional schizasterid species were obtained as ethanol-preserved specimens from various museum collections (Table 1). Species selection was aimed at complementing the list of schizasterid and selected outgroup taxa previously analyzed with regard to absence or presence of the IC (Ziegler, 2014).

**TABLE 1:** List of schizasterid species analyzed in the present study.

Species	Specimen information (locality; test length; source; method)
*Abatus cavernosus* (Philippi, 1845)	Kerguelen Islands, Indian Ocean; 27 mm; ZMB 5857; MRI Davis Station, Southern Ocean; 35 mm; unvouchered; dissection South Georgia, Southern Ocean; 38 mm; ZMH E7371; dissection Dundee Island, Southern Ocean; 45 mm; UB 2013.02.11.74; dissection
*Abatus cordatus* (Verrill, 1876)	Kerguelen Islands, Indian Ocean; 28 mm; ZSM 20011462; μCT Kerguelen Islands, Indian Ocean; 29 mm; ZMB 5437; MRI Kerguelen Islands, Indian Ocean; 33 mm; UB 2015.11.22.28; dissection Kerguelen Islands, Indian Ocean; 34 mm; NHMD; dissection Kerguelen Islands, Indian Ocean; 38, 41, 50 mm; UB 2015.12.8.42, 2015.11.22.28, 2015.11.22.28; dissection Kerguelen Islands, Indian Ocean; 42 mm; UB 2015.12.8.59; μCT + PTA Kerguelen Islands, Indian Ocean; four adults; UB 2015.11.28.14, 2015.11.28.18; NGS
*Brisaster antarcticus* (Döderlein, 1906)	Kerguelen Islands, Indian Ocean; 43 mm; NHMD; dissection Antipodes Island, Pacific Ocean; 51 mm, NIWA 114011; dissection Heard Island, Indian Ocean; 52 mm; AAD HIMI H171; dissection
*Brisaster capensis* (Studer, 1880)	Cape Peninsula, Atlantic Ocean; 48 mm; NHMD; dissection Cape Peninsula, Atlantic Ocean; 48 mm; ZMB 5855; dissection
*Brisaster fragilis* (Düben and Koren, 1844)	Tromsø, Atlantic Ocean; 25 mm; ZMB 2766; dissection Faroe Islands, Atlantic Ocean; 26 mm; NHMD; μCT Davis Strait, Atlantic Ocean; 30 mm; AM J.2630; dissection Bergen, Atlantic Ocean; 32 mm; ZSM 20011687; dissection Greenland, Atlantic Ocean; 45 mm; ZMH E7859; dissection Faroe Islands, Atlantic Ocean; 50 mm; NHMD; dissection
*Brisaster latifrons* (Agassiz, 1898)	Hokkaido, Pacific Ocean; 46 mm; MCZ 4407; dissection Sea of Japan, Pacific Ocean; 52 mm; UMUTZ Ecn-SI18-32; dissection Puget Sound, Pacific Ocean; 66 mm; unvouchered; dissection
*Brisaster moseleyi* (Agassiz, 1881)	Isla de los Estados, Atlantic Ocean; 55 mm; unvouchered; dissection
*Brisaster owstoni* (Mortensen, 1950)	Sagami Bay, Pacific Ocean; 18, 23, 26, 28, 29 mm; MMBS Ec551, Ec659, 1888, 1908, 1921; dissection
*Brisaster townsendi* (Agassiz, 1898)	Long Beach, Pacific Ocean; 21, 22, 23 mm; ZMB 7440, 7441, 7441; μCT + PTA Long Beach, Pacific Ocean; 27, 28, 29, 29, 29, 30, 31, 32, 33, 34, 35, 36, 37, 38, 40, 41, 41, 41, 42, 42, 42, 42, 42, 42, 42, 43, 44, 44, 44, 44, 45, 45, 45, 46, 46, 46, 47, 47, 47, 48, 48, 48, 48, 49, 49, 49, 50, 50, 50, 50, 51, 51, 51, 51, 51, 53, 53, 54, 55, 55, 55, 55, 56, 56, 57, 58, 58, 66 mm; unvouchered; dissection San Luis Obispo, Pacific Ocean; 51 mm; CASIZ 92728; dissection Long Beach, Pacific Ocean; 42, 46, 47 mm TL; unvouchered; TEM Long Beach, Pacific Ocean; three adults; unvouchered; SEM Long Beach, Pacific Ocean; 42–56 mm TL; unvouchered; NGS
*Genicopatagus affinis* (Agassiz, 1879)	Coronation Island, Southern Ocean; 51 mm; UB; dissection Coronation Island, Southern Ocean; 61 mm; UB; dissection
*Parapneustes cordatus* (Koehler, 1912)	Helmert Bank, Southern Ocean; 40 mm; NHMD; dissection Lyddan Island, Southern Ocean; 48 mm; UB; dissection
*Protenaster australis* (Gray, 1851)	Sydney Harbour, Pacific Ocean; 42 mm; unvouchered; dissection
*Protenaster rostratus* (Smith, 1878)	Queensland, Pacific Ocean; 28 mm; QM GL271; μCT + PTA
*Pseudabatus nimrodi* (Koehler, 1911)	Davis Station, Southern Ocean; 40 mm; unvouchered; dissection Davis Station, Southern Ocean; 52, 53 mm; unvouchered; dissection Dumont D’Urville Station, Southern Ocean; 59 mm; UB; dissection Cape Armitage, Southern Ocean; 64 mm; SIO E3079; dissection
*Schizocosmus abatoides* (Clark, 1925)	Weddell Sea, Southern Ocean; 33 mm; ZMH E7342; dissection Dundee Island, Southern Ocean; 50 mm; UB; dissection
*Tripylaster philippii* (Gray, 1851)	Arica, Pacific Ocean; 33 mm; SIO E831; dissection Antarctica, Southern Ocean; 39 mm; NHMD; dissection Argentina, Atlantic Ocean; 44 mm; ZMH E7774; dissection Locality unknown; 57 mm; MCZ 3235; dissection Southern Argentina, Atlantic Ocean; 60 mm; ZMH E7775; dissection
*Tripylus excavatus* (Philippi, 1845)	Southern Argentina, Atlantic Ocean; 37 mm; ZMH E4181; μCT + PTA

*AAD, Australian Antarctic Division; Kingston, TAS, Australia; AM, Australian Museum, Sydney, NSW, Australia; CASIZ, California Academy of Sciences, Invertebrate Zoology, San Francisco, CA, United States; MMBS, Misaki Marine Biological Station, Misaki, Japan; MCZ, Museum of Comparative Zoology, Cambridge, MA, United States; NHMD, Natural History Museum Denmark, Copenhagen, Denmark; NIWA, National Institute of Water and Atmospheric Research, Wellington, New Zealand; QM, Queensland Museum, Brisbane, QLD, Australia; SIO, Smithsonian Institution of Oceanography, San Diego, CA, United States; UB, Université de Bourgogne, Dijon, France; UMUTZ, University Museum, University of Tokyo, Department of Zoology, Tokyo, Japan; ZMB, Museum für Naturkunde, Berlin, Germany; ZMH, Zoologisches Museum Hamburg, Hamburg, Germany; ZSM, Zoologische Staatssammlung München, Munich, Germany.*

### Dissection and Photography

In total, 68 specimens of *B. townsendi* (27–66 mm test length, TL) were vivisected and their internal organs observed using a SZX10 stereomicroscope (Olympus Scientific Solutions Inc., Waltham, MA, United States) with attached D5300 digital camera (Nikon Inc., Melville, NY, United States). Preparations were carried out by removing the aboral half of the test together with all gonadal tissue. The internal organization of each animal as well as color of the IC and its contents were surveyed. In addition, ten ethanol-fixed specimens of *A. cordatus* as well as specimens of further schizasterid species were dissected in order to document absence or presence as well as shape and size of the IC.

### Micro-Computed Tomography

Three-dimensional (3D) analysis of internal organ systems was conducted using contrast-enhanced μCT. Three intact specimens of *B. townsendi* (21, 22, 23 mm TL) were formalin-fixed (4% in seawater) and, after several weeks of storage in fixative, were transferred to 70% ethanol through a graded series, and finally contrasted with 0.3% phosphotungstic acid (PTA) in 70% EtOH for 4 weeks (Ziegler et al., 2018; Ziegler, 2019). A single, ethanol-fixed specimen of *A. cordatus* (42 mm TL) as well as two formalin-fixed museum specimens (*Protenaster rostratus*, 28 mm TL and *Tripylus excavatus*, 37 mm TL) were treated in the same way. All contrast-enhanced samples were scanned using a SkyScan 1272 μCT system with a detector size of 4,032 × 3,280 px (Bruker microCT, Kontich, Belgium). In order to achieve higher isotropic voxel resolutions, a single intact IC from a formalin-fixed specimen of *B. townsendi* (22 mm TL) was excised together with adjacent digestive tract elements and scanned as well. For comparison, a single adult specimen each of *A. cordatus* (28 mm TL) and *Brisaster fragilis* (26 mm TL) was scanned using conventional μCT on a Phoenix Nanotom μCT system with a detector size of 2,304 × 2,304 px (GE Sensing & Inspection Technologies, Wunstorf, Germany). Reconstruction of the projection images was performed using the software provided with each scanner, i.e., NRecon 6.4 (Bruker microCT) and DatosX Reconstruction 1.5 (GE Sensing & Inspection Technologies). Visualization of two-dimensional (2D) and 3D data was carried out according to previously published protocols (Ziegler and Menze, 2013; Ziegler et al., 2018).

### Magnetic Resonance Imaging

MRI data for one intact museum specimen each of *A. cavernosus* (27 mm TL) and *A. cordatus* (29 mm TL) previously obtained using a Pharmascan 70/16 MRI system (Bruker Biospin GmbH, Ettlingen, Germany) were integrated into the present study. Information on specimen preparation for MRI and scanning parameters has been published elsewhere (Ziegler and Mueller, 2011; Ziegler et al., 2014).

### Morphometric and Volumetric Analyses

2D measurements (to nearest mm) of TL, width, and height as well as IC length and width (to the nearest 0.1 mm) were made using size-calibrated photographs gathered during specimen dissection. In several cases, test height was inferred using height-to-length ratios obtained through calibrated imagery of schizasterid test dimensions taken from the original species descriptions. Measurements were performed using Fiji/ImageJ 1.52s (Schindelin et al., 2012). Test volume (TV) was estimated by applying the geometric formula for an oblate spheroid, and IC volume (ICV) using the formula for a cylinder. The morphometric and volumetric values gathered using this approach were compared with 3D datasets obtained using MRI as well as μCT and were found to be congruent. The resulting data were plotted using Prism 8.3.1 (GraphPad Software Inc., San Diego, CA, United States).

### Light Microscopy

During vivisection, ICs of selected larger specimens of *B. townsendi* were excised and their contents extracted using a plastic pipette. The liquid was placed on a glass slide, covered, and immediately observed using a BX51 light microscope (Olympus Scientific Solutions Inc.) with attached QIClick monochrome digital video camera (Teledyne QImaging Inc., Surrey, BC, Canada). A representative video was edited using Windows 10 Video-Editor software (Microsoft Corp., Redmond, CA, United States) and saved as an MPEG-4 file. In addition, semi-thin sections (1 μm) of excised ICs were obtained on an EM UC6 ultramicrotome (Leica Microsystems, Wetzlar, Germany) from epoxy resin-embedded samples originally prepared for TEM. The semi-thin sections were placed on slides, stained using toluidine blue, covered, and imaged using a BX51 light microscope with attached CC-12 digital camera (Olympus Soft Imaging Systems GmbH, Münster, Germany).

### Scanning Electron Microscopy

For SEM, three adult specimens of *B. townsendi* were dissected immediately after collection while still on board the research vessel, and extracted ICs were fixed in 2.5% glutaraldehyde in Sorenson’s phosphate buffer at pH 7.2. Samples were held in fixative at 4°C for several days, then rinsed three times in the same buffer and postfixed in 1% OsO_4_ for 1 h. After the initial osmium treatment, they were rinsed once in buffer, once in buffer diluted to 50% with water, and once more in pure water. Tissues were then subjected to thiocarbohydrazide-mediated osmium binding (Kelley et al., 1975): they were immersed in a saturated solution of thiocarbohydrazide for 1 h, rinsed twice with water, then treated with 1% OsO_4_ in water for 1 h. Tissues were then rinsed twice in water, dehydrated through an ascending ethanol series (to 100%), critical-point-dried using a Samdri-PVT-3D system (Tousimis Research Corp., Rockville, MD, United States) with CO_2_ as the transitional fluid, and mounted on stubs using copper-conductive tape with conductive adhesive on both sides. Specimens were coated with gold/palladium using a Pelco SC-4 sputter-coater (Ted Pella Inc., Redding, CA, United States) and imaged with a FEI Quanta 200 scanning electron microscope (Thermo Fisher Scientific Inc., Waltham, MA, United States).

### Transmission Electron Microscopy

For TEM, three specimens of *B. townsendi* (42, 46, 47 mm TL) were dissected immediately after collection while still on board the research vessel, and extracted ICs as well as pieces of intestinal wall were fixed in 2.5% glutaraldehyde in Sorenson’s phosphate buffer (pH 7.2). Fixed samples were held at 4°C for several days, then rinsed three times in buffer and postfixed in 1% OsO_4_ in buffer for 1 h. After osmium treatment, they were rinsed three times in buffer, dehydrated through an ethanol series of ascending concentrations (to 100%), transferred to propylene oxide, infiltrated, and then embedded in Araldite 502 epoxy resin (Electron Microscopy Sciences, Hatfield, PA, United States). Ultrathin sections (70 nm) were obtained with a diamond knife using an EM UC6 ultramicrotome (Leica Microsystems). Sections were mounted on Formvar-covered copper grids, stained with uranyl acetate and lead citrate, and examined using an EM 10 CR transmission electron microscope (Carl Zeiss AG, Oberkochen, Germany). Electron micrographs were digitally recorded from phosphor imaging plates using a Micron imaging plate scanner (DITABIS, Pforzheim, Germany). Composite overview images were created using the software AutoStitch (Brown and Lowe, 2007).

### Next Generation Sequencing

For molecular inferences, ICs from 12 specimens of *B. townsendi* (42–56 mm TL) were extracted following dissection on the research vessel. The organs and their contents were each placed into sterile plastic vials stored on ice; within 3 h of collection they were transferred to -80°C for storage until analysis. For two of these specimens (43, 48 mm TL), small pieces of stomach, intestine, and rectum (and their contents) were also sampled and treated in the same way. ICs from *A. cordatus* were extracted from four adult specimens that had been directly fixed in 95% ethanol following collection. For all samples, nucleic acids were extracted using the FastDNA SPIN Kit for Soils (MP Biomedicals, Solon, OH, United States). The success of extractions was confirmed via gel electrophoresis (1% agarose) and quantified through comparison with the 1 kb exACTGene DNA ladder (Fisher Bioreagents, Pittsburgh, PA, United States).

Extracted samples were sequenced using a MiSeq NGS system with the Reagent Kit V2 (Illumina, San Diego, CA, United States) to generate paired-end sequences. The V4 variable regions of 16S rRNA genes were amplified using the barcoded primer pair 515F-806R (Apprill et al., 2015; Parada et al., 2016). Negative PCR controls were run during the sequencing reaction and none yielded positive results. The reaction mixture and thermocycling specifications were carried out in accordance with rules set forth by the Earth Microbiome Project (Caporaso et al., 2011). Analyses of microbiome data were carried out using QIIME 2 2016.8 (Bolyen et al., 2019). Raw sequence data were demultiplexed and quality-filtered using the *q2-demux* plugin followed by denoising with DADA2 via *q2-dada2* (Callahan et al., 2016), which included removing singleton sequences. The minimum sequence length found by DADA2 was 150 base pairs (bp), and the maximum was 254 bp, while the minimum threshold for quality control was 150 bp using a Phred quality score to represent confidence in assignment of the base call by the Illumina sequencer. Most sequences maintained a score above 30 until the 151 bp mark. Amplicon sequence variants (ASVs) were aligned with MAFFT via *q2-alignment* (Katoh et al., 2002). Aligned ASVs were then used in construction of a phylogeny using fasttree2 via *q2-phylogeny* (Price et al., 2010). Alpha diversity metrics in the form of a Shannon’s diversity index and operational taxonomic units (OTUs), beta diversity metrics in the form of Bray-Curtis and unweighted UniFrac dissimilarity (Lozupone and Knight, 2005), as well as Principal Coordinates Analysis (PCoA) were estimated via *q2-diversity* for each sample and visualized using the EMPeror software package (Vázquez-Baeza et al., 2013).

Taxonomy was assigned to the ASVs via *q2-feature-classifier* (Bokulich et al., 2018) using a naïve Bayesian taxonomy classifier against the SILVA 97% database (Yilmaz et al., 2014). Kruskal-Wallis statistical comparisons for alpha diversity metrics and PERMANOVA beta diversity comparisons of unweighted UniFrac dissimilarity were made in QIIME 2 via *alpha-group-significance* and *beta-group-significance*, respectively. Differential abundance analysis was performed and visualized using a *feature-table* heatmap (Hunter, 2007) generated in QIIME 2 using the Bray-Curtis metric and UPGMA hierarchical clustering methods. As the initial numbers of taxa were highly complex, the visualizations were limited to the most abundant taxa (i.e., ≥ 2,000 OTUs for *B. townsendi* and ≥ 150 OTUs for *A. cordatus*) and the feature table was collapsed to level 5 taxonomy (i.e., family level) for *B. townsendi* and level 4 taxonomy (i.e., order level) for *A. cordatus*.

## Results

### Morphology of the Intestinal Caecum in *Brisaster townsendi* and *Abatus cordatus*

**FIGURE 1:**
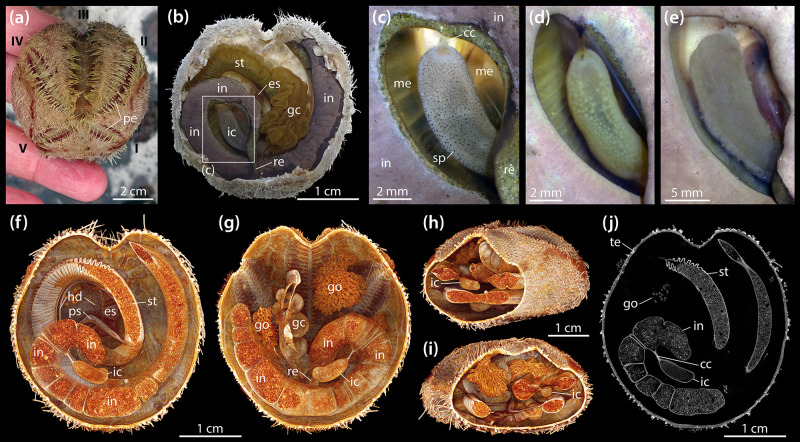
Morphology of the intestinal caecum (IC) in *Brisaster townsendi*. **(a)** Aspect of a living animal, aboral view; Roman numerals denote ambulacra. **(b)** Situs of vivisected specimen with aboral part of test and attached gonadal material removed, aboral view. **(c–e)** Close-up photographs of ICs from three different specimens, aboral views. **(f–i)** Volume renderings of a μCT dataset with 9.5 μm isotropic voxel resolution: virtual slicing of the renderings in aboral **(f)** and oral **(g)** as well as oblique posterior **(h)** and anterior **(i)** views. **(j)** Virtual horizontal section of the dataset at the level of the IC. cc, caecal canal; es, esophagus; gc, gastric caecum; go, gonad; hd, haemal duct; ic, intestinal caecum; in, intestine; me, mesentery; pe, petal; ps, primary siphon; re, rectum; sp, spot; st, stomach; te, test.

Adults of *B. townsendi* have two relatively short posterior petals and a deeply sunken petal in ambulacrum III (Figure 1a). The digestive tract shows the typical spatangoid form and is composed of a predominantly tubular gut that can be divided into esophagus, stomach, intestine, and rectum (Figure 1b). As in all other heart urchins, pharynx and Aristotle’s lantern are absent. The entire digestive tract is suspended from the relatively thin calcareous test by mesenteric strands, the outer ones of which are fenestrated. In addition to the four main gut compartments, the digestive tract also possesses a tubular primary and secondary siphon laterally bypassing the anterior part of the stomach, a large and convoluted gastric caecum attached to the aboral side of the anterior part of the stomach, and a prominent IC. The IC is a kidney-shaped, almost cylindrical, blind-ending sac (Figure 1b) laterally connected to the posterior part of the intestine through the short caecal canal (Figure 1c). The organ is located within a coil formed by the posterior part of the intestine and the rectum. In living specimens, the wall of the IC is semi-transparent and may bear dark (Figure 1c) or bright spots (Figure 1d), or no spots at all (Figure 1e). Seen through its semi-transparent wall, the contents of the organ either appear as a partially dark (Figures 1c,d) or bright mass (Figure 1e). In a subset of vivisected specimens (*N* = 50), 38 (76%) of the ICs had no, 6 (12%) had dark, and 6 (12%) had bright spots, while 31 (62%) had dark and 19 (38%) had bright contents.

In contrast to esophagus and stomach, the intestine and IC do not bear any large haemal ducts (Figures 1f,g). Due to its suspension from an inner mesentery that spans the posterior gut coil, the IC is surrounded by the coelomic fluid contained within the main perivisceral cavity, the somatocoel (Figures 1h,i). The IC is present both in male and female specimens and no sexual dimorphism in its form was observed. A virtual, μCT-based horizontal section through the IC of *B. townsendi* at the level of the caecal canal shows that the organ’s contents are homogeneous, while the contents of stomach and intestine are heterogeneous (Figure 1j). The findings on the organization of the digestive tract in *B. townsendi* also apply to *A. cordatus*.

### Anatomy of the Intestinal Caecum in *Brisaster townsendi*

**FIGURE 2:**
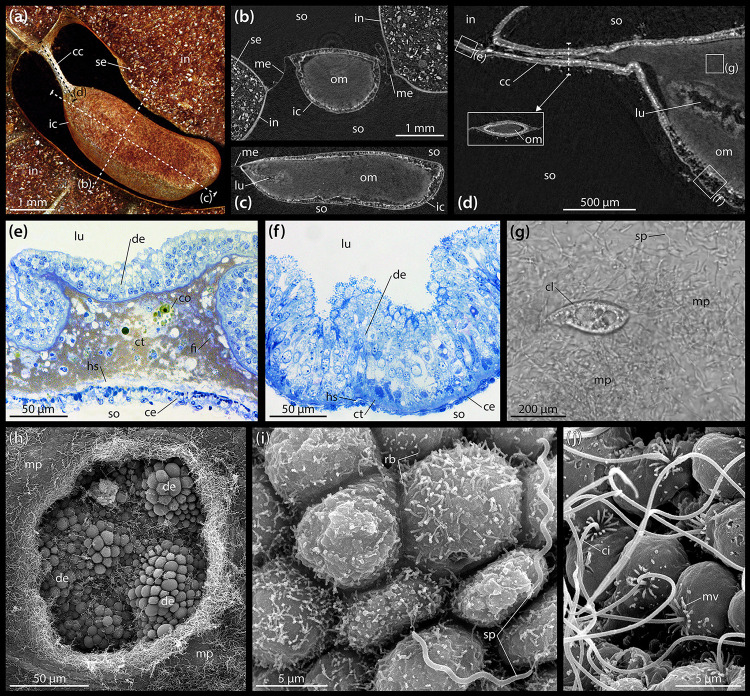
Anatomy and ultrastructure of the intestinal caecum (IC) in *Brisaster townsendi*. **(a)** Volume rendering of the IC and adjacent intestine based on a μCT scan with 9.5 μm isotropic voxel resolution, aboral view. **(b)** Virtual vertical section through the IC. **(c)** Virtual sagittal section through the IC. **(d)** Virtual sagittal section through the anterior part of the IC using a μCT dataset with 600 nm isotropic voxel resolution. **(e)** LM micrograph of a semi-thin section of the intestinal wall. **(f)** LM micrograph of a semi-thin section of the IC’s wall. **(g)** LM micrograph of the extracted contents of the IC showing microbial population and ciliate. **(h)** SEM micrograph of the microbial population inside the IC and the underlying digestive epithelium; the hole in the bacterial mass was artificially made. **(i)** SEM micrograph of the digestive epithelium lining the interior of the IC showing smaller, rod-shaped bacteria attached to cell apices as well as longer, spiral-shaped taxa free within the lumen. **(j)** SEM micrograph of the coelomic epithelium lining the exterior of the IC. cc, caecal canal; ce, coelomic epithelium; ci, cilium; cl, ciliate; co, coelomocyte; ct, connective tissue; de, digestive epithelium; fi, fibrocyte; hs, haemal space; ic, intestinal caecum; in, intestine; lu, lumen; me, mesentery; mp, microbial population; mv, microvillus; om, organic mass; rb, rod-shaped bacterium; se, sediment; so, somatocoel; sp, spiral-shaped bacterium.

A closer look at the gut contents reveals that the heterogeneous material observed within the esophagus, stomach, intestine, and rectum is primarily composed of sediment (Figure 2a), while the largely homogenous contents of the IC are organic, as can be inferred from their staining properties under contrast-enhanced μCT (Figures 2b,c). These observations also hold true in *A. cordatus*. At the anterior end of the IC, a centrally located, small lumen may be observed that bulges inward toward the center of the organ from the caecal canal (Figure 2d). While individual sediment grains inside the intestine can readily be discerned at 9.5 μm isotropic voxel resolution (Figures 2a,b), the contents of the IC cannot be differentiated in this as well as another scan acquired at 600 nm isotropic voxel resolution (Figure 2d). As the virtual section through this latter dataset shows, the caecal canal connecting the IC to the intestine is partially filled with the organic mass contained within the organ (Figure 2d). However, no sphincter or lip was found in the vicinity of the caecal canal that would prevent sediment from entering the IC.

The histology of the digestive tract wall follows the basic spatangoid pattern. From the gut lumen toward the somatocoel, an inner digestive epithelium is bordered by a middle connective tissue layer and finally an outer coelomic epithelium. In the intestine, the digestive epithelium is heavily folded (Figure 2e). The enterocytes of the digestive epithelium are simple columnar cells that measure about 30–40 μm in height and 5–10 μm in width. The connective tissue layer is well developed, ranging from 20 to 100 μm in thickness. Large parts of the extra-cellular matrix (ECM) are filled by a golden-brown granular material with intermittent coelomocytes and occasionally fibrocytes. In close proximity to the coelomic epithelium, the connective tissue layer contains a relatively wide haemal space of about 10–20 μm height. The coelomic epithelium itself exhibits a height of about 10–20 μm and is composed of a pseudostratified epithelium consisting of a basal layer of myoepithelial and a top layer of squamous epithelial cells each of about 5–10 μm in height and 5–10 μm in width. Seen from the somatocoelomic side, the outer wall of the intestine appears relatively smooth (Figure 1b).

The histological properties of the IC’s wall differ significantly from those of the intestine. The digestive epithelium is arranged in irregular bumps separated by depressions and which measure about 50 μm in height and 100 μm in width (Figure 2f). As in the intestine, the enterocytes of the digestive epithelium are simple columnar cells, but here they measure about 70–90 μm in height and 5–10 μm in width. The connective tissue layer is weakly developed, with a thickness of 5–10 μm. The relatively small haemal space measures about 2–5 μm in height. The coelomic epithelium is characterized by a single layer of squamous cells with about 5–10 μm height and 5–10 μm width (Figure 2f). Seen from the somatocoelomic side, the outer wall of the IC appears entirely smooth (Figures 1c–e).

LM observations of the organic mass inside the IC obtained from living specimens show that the organ contains a microbial population composed of motile spirochetes each about 10 μm in length, as well as smaller spiral- and rod-shaped bacteria (Supplementary Video S1). In addition, ciliates can occasionally be observed (Figure 2g).

### Ultrastructure of the Intestinal Caecum in *Brisaster townsendi*

SEM investigations reveal that the IC’s digestive epithelium is covered by a dense microbial population (Figure 2h). As also observed with LM (Figure 2g), the most obvious component of this bacterial mass are spirochetes that occur within the lumen of the IC and are not attached to the apical side of the digestive epithelium. Instead, the apices of the enterocytes are predominantly covered by smaller, rod-shaped bacteria (Figure 2i). The coelomic epithelial cells of the IC possess a collar of microvilli surrounding a single, long cilium (Figure 2j). In contrast to the digestive epithelial cells, the coelomic epithelial cells are not covered by a dense fringe of bacteria.

**FIGURE 3:**
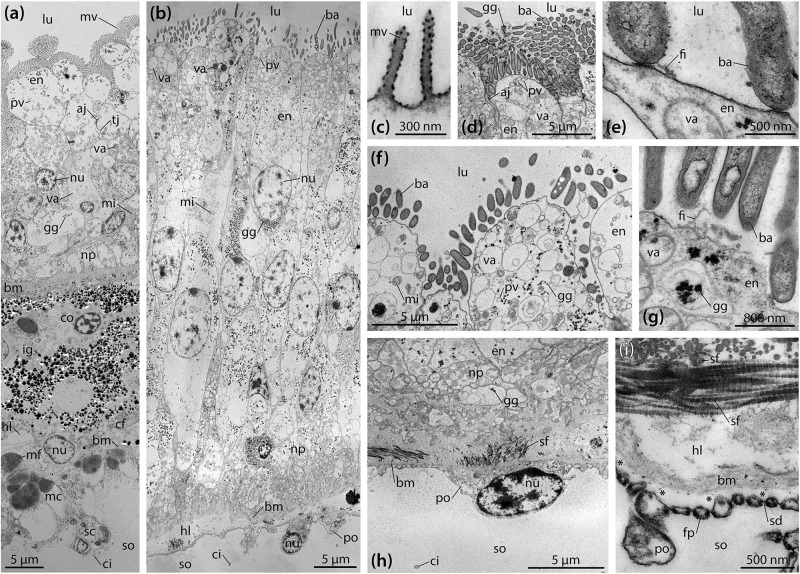
Ultrastructure of the intestine and intestinal caecum (IC) in *Brisaster townsendi*. **(a,b)** Composite overview images showing a vertical section through the intestinal **(a)** and the IC’s wall **(b)**. **(c–i)** Structural features of the IC’s wall. **(c)** Tiny microvilli at the enterocytes’ apices. **(d)** Numerous rod-shaped bacteria attached to the digestive epithelium of the IC. **(e)** Attachment site of rod-shaped bacteria. **(f)** Pinocytotic vesicles and vacuoles in the apical area of the enterocytes. **(g)** Glycogen granules near the apex of the enterocytes. **(h)** Podocyte of the coelomic epithelium with underlying connective tissue and folds of the digestive epithelium. **(i)** Podocyte’s foot processes bordering the basement membrane with underlying haemal lacunae and striated collagen fibrils. Asterisks denote artificial detachment of the coelothel from the basement membrane. aj, adherens junction; ba, bacterium; bm, basement membrane; cf, collagen fibril; ci, cilium; co, coelomocyte; en, enterocyte; fi, filamentous organism; fp, foot process; gg, glycogen granule; hl, haemal lacuna; ig, iron granule; lu, lumen; mc, myocyte; mf, myofibril; mi, mitochondrion; mv, microvillus; np, nerve plexus; nu, nucleus; po, podocyte; pv, pinocytotic vesicle; sc, squamous cell; sd, slit diaphragm; sf, striated fibril; so, somatocoel; tj, tight junction; va, vacuole.

TEM analyses show that the digestive epithelium of the intestine is characterized by simple, non-glandular enterocytes that do not secrete mucous (Figure 3a). Apically, these non-ciliated enterocytes possess a dense zone of unbranched microvilli measuring about 1.75 × 0.15 μm, but no terminal web was observed. The non-muscular enterocytes join apically through upper adherens and lower tight junctions. In the apical region, numerous pinocytotic vesicles and electron-dense as well as clear vacuoles can be seen inside the cytoplasm. The centrally to basally located nuclei are subspherical and measure about 4.5 × 3.5 μm. A Golgi apparatus is present lateral or apical to the nucleus, while cisternae of the rough endoplasmic reticulum (RER) were rarely found. Glycogen granules and small, subspherical mitochondria can be seen scattered throughout the enterocytes. The epineural basiepithelial nerve plexus can be found near the basal region of the digestive epithelium. The base of the enterocytes is smooth and rests upon a basement membrane of about 2 μm thickness. The ECM is interspersed with coelomocytes and fibrocytes. In addition, numerous electron-dense granules measuring about 0.1–1.2 μm in diameter can be found scattered within the ECM. These granules appear to increase in size from digestive to coelomic epithelium. A loose zone of elastic-like collagen fibers is located at the borders of the ECM and the basement membrane of digestive and coelomic epithelium. Numerous haemal lacunae occur within the loose zone of the ECM. The coelomic epithelium of the intestine rests on a basement membrane of about 200 nm thickness. The hyponeural basiepithelial nerve plexus is present between the cells of the lower layer of the pseudostratified coelomic epithelium. The basally located myoepithelial cells predominantly possess longitudinal myofibrils. The squamous upper cells have ovoid nuclei measuring about 5 × 3 μm and scattered spherical mitochondria as well as frequent glycogen granules, while RER cisternae are few. The covering cells have adherens junctions apically and are characterized by the presence of a collar of microvilli surrounding a single cilium.

Like the intestine, the digestive epithelium of the IC is characterized by simple, non-glandular enterocytes that do not secrete mucous (Figure 3b). At their apex, these non-ciliated enterocytes occasionally possess small microvilli measuring about 400 × 80 nm (Figure 3c). However, the most prominent feature here is a dense fringe of bacteria that adhere to the apical membrane (Figures 3b,d–g). The non-muscular enterocytes are joined apically through adherens junctions (Figure 3d). In the enterocytes’ apical region, numerous pinocytotic vesicles and clear as well as electron-dense vacuoles occur within the cytoplasm (Figure 3f). However, no endocytosis of bacteria was observed. The centrally located nuclei are elliptical in shape and measure about 10 × 5 μm, their long axis oriented along the enterocytes’ main axis (Figure 3b). No prominent Golgi apparatus or RER cisternae are present. However, numerous glycogen granules and subspherical as well as elongated mitochondria are distributed throughout the enterocytes (Figures 3b,d–g). A well-developed epineural basiepithelial nerve plexus can be found near the base of the digestive epithelium (Figure 3h). The enterocytes are strongly folded at their base, interdigitating through numerous protrusions with a basement membrane of about 3–4 μm thickness. The underlying ECM is characterized by a haemal space of about 2–10 μm thickness and is interspersed with coelomocytes and occasionally fibrocytes. A dense zone of circular and longitudinal striated collagen fibrils each measuring 40–80 nm in diameter borders the ECM and the basement membrane of the coelomic epithelium (Figure 3h). The coelomic epithelium of the IC rests upon a basement membrane of about 100–400 nm thickness. The relatively inconspicuous hyponeural basiepithelial nerve plexus is present between the cells of the coelomic epithelium (Figure 3h). The cells of the coelomic epithelium are podocytes, but only a small number of myofibrils can be observed in these cells. The podocytes possess subspherical nuclei measuring about 5 × 3 μm and scattered mitochondria as well as glycogen granules, while RER cisternae are few. The somata of the podocytes measure about 8 × 4.5 μm (Figure 3h). In cross-section, their pedicels have dimensions of 350–450 nm in height and 250–350 nm in width, while the foot processes measure 100–200 nm in height and 100–250 nm in width. The slit diaphragms between individual pedicels and foot processes are about 20–30 nm wide (Figure 3i). Similar to the intestine, a collar of microvilli surrounds a single cilium on each coelomic epithelial cell, as also observed with SEM (Figure 2j).

### The Gut Microbiome of *Brisaster townsendi* and *Abatus cordatus*

**FIGURE 4:**
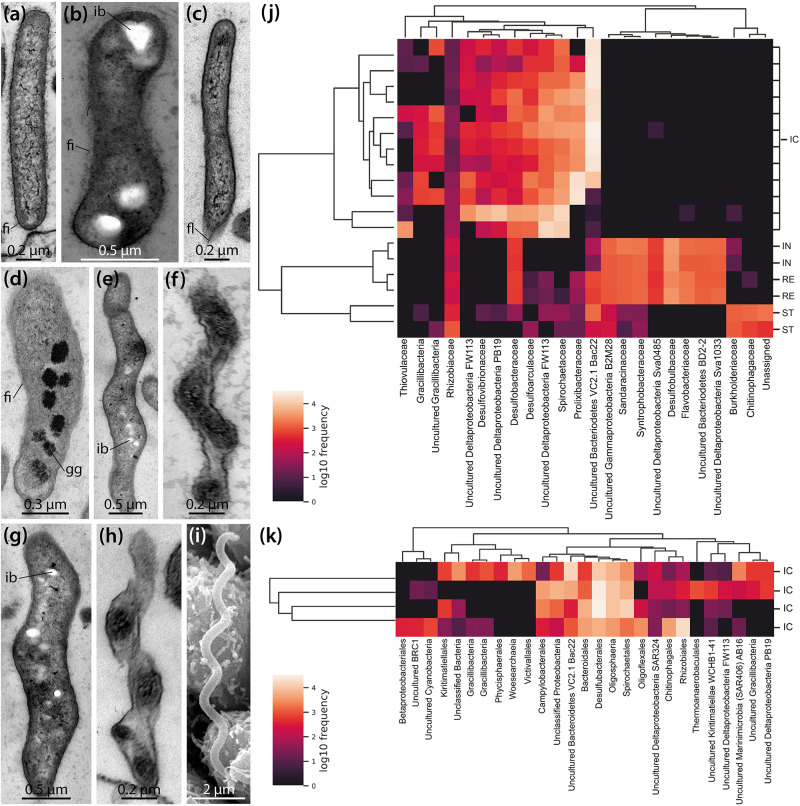
Ultrastructural and genomic analyses of the microbial population found inside the intestinal caecum (IC) as well as selected gut compartments of *Brisaster townsendi* and *Abatus cordatus*. **(a–i)** TEM and SEM micrographs showing different bacterial morphotypes found inside the IC of *B. townsendi*. **(j,k)** Heatmaps depicting co-occurrence of the most abundant operational taxonomic units (OTUs) across four gut compartments with a minimum OTU frequency of 2,000 in *B. townsendi* **(j)** and from ICs with a minimum OTU frequency of 150 in *A. cordatus* **(k)**; colored scales show relative abundance of taxa across all samples, clusters on the left depict Bray-Curtis metric across samples and on the top depict UPGMA hierarchical clustering, SILVA taxonomic identifications were collapsed to family level **(j)** and order level **(k)** or higher if taxonomic classification to those levels was not possible. fi, fimbria; fl, flagellum; gg, glycogen granule; ib, inclusion body; ic, intestinal caecum; in, intestine; re, rectum; st, stomach.

LM, SEM, and TEM observations show that a variety of bacterial morphotypes occur inside the IC of *B. townsendi* (Figures 2g–i, 3d–g). These include straight or curved rod-shaped bacteria measuring from about 2.0 × 0.2 to 3.0 × 0.5 μm (Figures 4a–d), small spiral-shaped bacteria measuring from about 1.5 × 0.2 to 5.0 × 0.5 μm (Figures 4e–h), and larger spiral-shaped bacteria measuring about 9.5 × 0.4 μm (Figure 4i). No bacterial stacks, spores, capsules, or buds are observed, but occasionally small filamentous structures measuring about 1.0 × 0.1 μm can be seen (Figures 3e,g). These as well as the rod-shaped bacteria are usually found on or near the apical side of the IC’s digestive epithelium (Figures 3d–g), while the spiral-shaped bacteria are predominantly found free within the organ’s lumen (Figures 2g–i and Supplementary Video S1). The majority of organisms directly attached to the enterocytes are rod-shaped bacteria (Figure 4a), although spiral-shaped bacteria can occasionally be found attached to the digestive epithelium as well (Figure 4h). At the attachment sites of these two bacterial morphotypes to the enterocytes there is no sign of direct cytoplasmic interaction between host and bacterial cells (Figures 3e, 4a,h). Additional bacterial forms include curved rod-shaped bacteria with (Figure 4b) or without (Figure 4c) a dense cover of fimbriae as well as spiral-shaped taxa without any apparent fimbriae (Figures 4e–i). Some of the rod- as well as the spiral-shaped morphotypes have at least one polar flagellum (Figure 4c). In addition, some bacteria show clear inclusion bodies (Figures 4b,e,g). More frequently, glycogen granules are seen between the dense fringe of bacteria adhering to the enterocytes’ apex (Figure 3d) and sometimes these granules are also observed inside bacteria (Figure 4d). Where the IC lumen contains a substantial amount of glycogen granules, the adjacent digestive epithelium appears to show increased pinocytotic activity (Figure 3d).

To complement these structural findings, a total of 855,592 16S rRNA gene sequences were generated from four regions of the digestive tract (stomach, intestine, IC, and rectum) in *B. townsendi* as well as from the IC in *A. cordatus*, representing 2,738 OTUs across all samples. The overwhelming majority of OTUs in all gut compartments were identified as bacterial, with only a single nanoarchaeal lineage; this latter OTU was observed in all four *A. cordatus* IC samples, but not in any *B. townsendi* samples (Supplementary Tables S1, S2). The OTUs in *B. townsendi* and *A. cordatus* IC samples showed limited overlap at the 97% similarity cut-off, which roughly corresponds to species level. However, when viewed at a higher taxonomic level, there was more similarity as > 50% of the OTUs in both species’ IC microbiomes were members of the Bacteroidetes, sulfate-reducing Deltaproteobacteria lineages, and Spirochaetes. The most abundant taxon in most of the ICs of *B. townsendi* was most closely related to a yet uncultured member of the Bacteroidetes, the VC2.1 Bacc22 group (Supplementary Table S1). The most abundant taxon in ICs of *A. cordatus* was an unknown species of Gracilibacteria (Supplementary Table S2), which was present, but at lower abundance, in ICs of *B. townsendi* as well.

The most abundant OTUs (i.e., ≥ 2,000 representatives) in the *B. townsendi* IC samples showed little to no overlap with the other gut compartments. This resulted in clear splits in the hierarchical clustering (Figure 4j), clearly distinguishing taxa found in the IC samples of *B. townsendi* from those in the other three gut compartments of that species. Only a single OTU, identified as a yet unknown species of *Mesorhizobium* (Supplementary Table S1), showed meaningful overlap between the IC and the other gut compartments (14% frequency in the IC vs. 86% frequency in the rest of the gut). While the total diversity of IC microbiomes in both species was primarily composed of Gram-negative, rod- or spiral-shaped, motile, obligately anaerobic taxa (e.g., *Desulfoconvexum, Sulfurimonas, Sediminispirochaeta*), members of the bacterial candidate phylum Gracilibacteria were also present, often in high numbers of amplicons. The relatively large sample size of ICs from *B. townsendi* (*N* = 12) and *A. cordatus* (*N* = 4) enabled us to examine intraspecific variation among IC microbial communities as well: substantial intraspecific variation was observed in both schizasterid species (Figures 4j,k and Supplementary Tables S1, S2).

**FIGURE 5:**
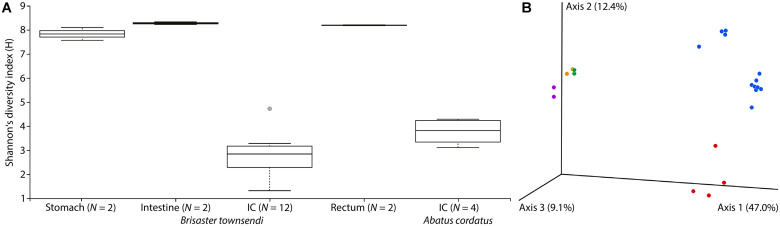
Microbial diversity in four gut compartments of *Brisaster townsendi* as well as in the intestinal caecum (IC) of *Abatus cordatus*. **(A)** Shannon’s diversity index plot for three main gut compartments and the IC of *B. townsendi* as well as the IC of *A. cordatus*; *p* = 0.004. **(B)** 3D unweighted UniFrac EMPeror PCoA plot of gut samples from *B. townsendi* shown in purple (stomach), orange (intestine), blue (IC), and green (rectum) as well as samples from *A. cordatus* shown in red (IC).

We also noticed major differences in community structure of the IC microbiome compared to other gut compartments. The IC microbiomes of *B. townsendi* and *A. cordatus* displayed much lower overall Shannon’s diversity indices than did the microbiomes of stomach, intestine, and rectum of *B. townsendi* (Figure 5A). The *B. townsendi* IC microbial communities were much less diverse than those of the other three *B. townsendi* gut compartments (*p* < 0.03 for all pairwise comparisons), and were relatively similar among all twelve sampled individuals. Similar patterns were observed for the number of OTUs, with an average of 69.8 (SD 30.9) in the *A. cordatus* and 61.9 (SD 12.1) in the *B. townsendi* IC samples compared to 660 (SD 38.2) in the stomach, 857.5 (SD 7.8) in the intestine, and 797.5 (SD 20.5) in the rectum of *B. townsendi*. Beta diversity comparisons confirmed that microbial species composition varied significantly across these five sets of samples [PERMANOVA, 999 permutations, pseudo-*F*_(4, 17)_ = 8.782, *p* = 0.001]. In addition, pair-wise comparisons showed that beta diversity of the *B. townsendi* IC samples was significantly different from all other digestive tract compartments as well as from the *A. cordatus* IC samples (*q* < 0.05 for all). No significant differences were observed in comparisons between each of the three main gut compartments, i.e., stomach, intestine, and rectum (*q* > 0.3 for all).

PCoA analyses using unweighted UniFrac and Bray-Curtis datasets were visualized in the first three dimensions. Both plots revealed similarly distinct separation among sample types, so only the UniFrac results are shown here (Figure 5B). Axis 1 explained roughly half (47.0%) of the variability in the data and clearly distinguished the two species’ IC microbiomes from the other gut compartments of *B. townsendi*. The second axis (12.4%) distinguished the IC samples from *A. cordatus* and *B. townsendi*, while the third axis (9.1%) highlighted differences among stomach, intestine, and rectum samples, which were mostly overlapping. In addition, two main clusters were observed within the IC samples of *B. townsendi* (Figure 5B), further highlighting the significant variability among these samples despite their identical provenance.

### Distribution, Shape, and Volume of the Intestinal Caecum in the Schizasteridae

**FIGURE 6:**
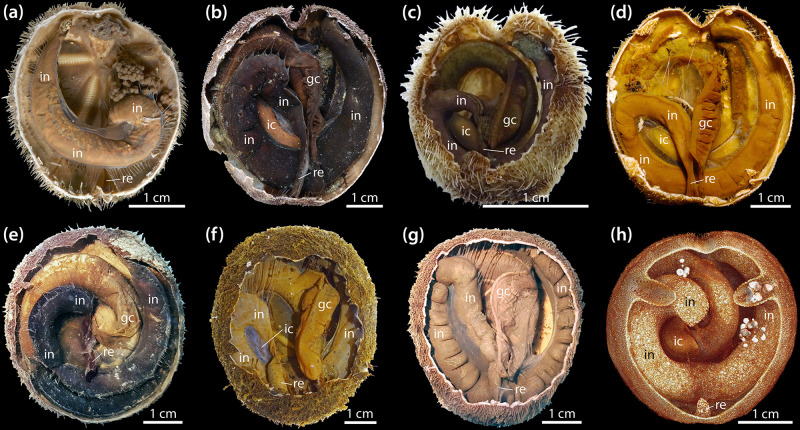
Absence and presence of the intestinal caecum (IC) in selected schizasterid taxa; all images show results obtained using fixed specimens. **(a)** *Protenaster australis*, oral view. **(b)** *Brisaster moseleyi*, aboral view. **(c)** *Brisaster owstoni*, aboral view. **(d)** *Brisaster townsendi*, aboral view. **(e)** *Genicopatagus affinis*, aboral view. **(f)** *Pseudabatus nimrodi*, aboral view. **(g)** *Schizocosmus abatoides*, aboral view. **(h)** *Tripylus excavatus*, aboral view of a sliced volume rendering obtained using a contrast-enhanced μCT dataset with 10 μm isotropic voxel resolution. gc, gastric caecum; ic, intestinal caecum; in, intestine; re, rectum.

Building on a study of several in- and outgroup taxa analyzed with regard to absence or presence of the IC (Ziegler, 2014), we examined additional species to provide a more comprehensive overview of the distribution of the IC among the Schizasteridae (Table 1). The IC is absent in the two extant species of the plesiomorphic schizasterid *Protenaster* (Figure 6a). An IC can now be confirmed to occur in all extant species of *Brisaster*, including *B. moseleyi* (Figure 6b), *B. owstoni* (Figure 6c), and *B. townsendi* (Figures 1, 6d). The IC is absent in the only extant species of *Genicopatagus*, *G. affinis* (Figure 6e), but present in *Pseudabatus nimrodi* (Figure 6f). In addition, the organ is absent in *Schizocosmus abatoides* (Figure 6g), but present in the single extant species of *Tripylus*, *T. excavatus* (Figure 6h). Where present, the IC is always found to be suspended from the mesentery spanning the posterior gut coil. Based on contrast-enhanced μCT data, the contents of the IC were found to be homogenous in *A. cordatus* and *T. excavatus*, as also observed for *B. townsendi* (Figures 1j, 2b–d).

In *Brisaster*, the IC is generally present in the form of a largely cylindrical, kidney-shaped structure (Figures 1f–i). This shape can be observed both in fresh as well as in formalin- or ethanol-fixed specimens (Figures 1b, 6d). While the IC takes this form in most species known to possess the organ, a few taxa differ in this regard. For example, the IC in *T. excavatus* is more rectangular in shape (Figure 6h), while a significant deviation from the general morphological pattern observed in *Brisaster* can be observed in *P. nimrodi*, where the organ is present as a reduced, elongated structure (Figure 6f).

**FIGURE 7:**
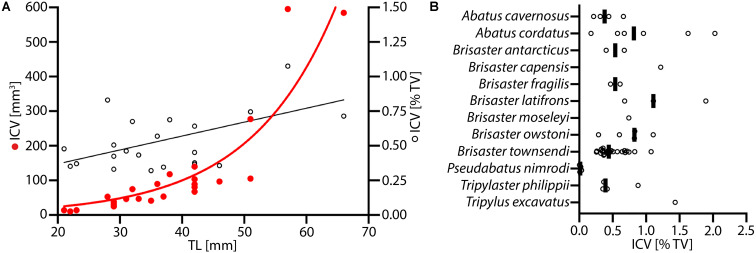
Volumetric data on the intestinal caecum (IC) in selected schizasterid taxa. **(A)** Size series of *Brisaster townsendi* (*N* = 24); red dots: IC volume (ICV) vs. test length (TL); black tori: ICV as function of test volume (TV) vs. TL; red line: exponential curve with *R*^2^ = 0.823 and *Y* = 5.491^(0.07255*X)^; black line: least squares regression with *p* < 0.0001, *R*^2^ = 0.418, and *Y* = 0.01005X + 0.1686. **(B)** ICV as function of TV in samples from twelve different schizasterid species, including *B. townsendi*; each data point is shown, and black bars represent mean values for each species.

Data on the ICV for specimens of *B. townsendi* ranging from 22 to 67 mm TL reveal an exponential increase in ICV as a function of TL, and a linear increase in the percentage of TV occupied by the IC as a function of TL (Figure 7A). Calculations of ICV and TV among different species show that on average the ICV forms about 0.5–1% of TV (Figure 7B). As can be expected from the morphological findings, the ICV is much smaller as a function of TV in *P. nimrodi* (0.025%) and much larger in *T. excavatus* (1.5%), while on average few differences in ICV are observed among species of *Brisaster* and *Abatus* (Supplementary Table S3). For example, the ICV of *B. townsendi* and *A. cordatus* does not differ much in animals of comparable size: 14 μl (23 mm TL) vs. 14 μl (29 mm TL), 103 μl (42 mm TL) vs. 121 μl (42 mm TL), and 584 μl (66 mm TL) vs. 593 μl (50 mm TL), respectively.

## Discussion

Due to their deposit-feeding habits and frequent high densities, schizasterid heart urchins can have significant effects on the ocean floor and on biogeochemical fluxes between sediment and overlying water (Chesher, 1963; Brownell, 1970; Thompson et al., 1987; Bromley et al., 1995; Féral and Poulin, 2011; Macdonald et al., 2012). However, despite their ecological importance, little is known of schizasterid digestive processes, their gut microbiomes, or the function of specialized digestive organs. To provide further insights into the microorganisms inhabiting the schizasterid digestive tract, we undertook a multimodal analysis of the gut of *Brisaster townsendi*. Members of the genus *Brisaster* are particularly suitable for study because of their established taxonomy, broad geographic distribution, and well-documented lifestyle (Mortensen, 1951; Ghiold, 1988-89; Kanazawa, 1992; Hood and Mooi, 1998; Walker and Gagnon, 2014). Most importantly, all species of *Brisaster* are characterized by the presence of the IC (Ziegler, 2014, this study), an enigmatic organ potentially harboring a distinct gut microbiome of unknown capacity.

### The Schizasterid Intestinal Caecum, a Highly Specialized Gut Compartment

While the presence of a hindgut diverticulum in selected species of *Brisaster* and *Abatus* had been known for some time (Brownell, 1970; De Ridder et al., 1985; Penry and Jumars, 1987; Plante and Jumars, 1992; De Ridder, 1994; Bromley et al., 1995; Schatt and Féral, 1996; Mayer et al., 1997), the IC was only recently shown to be an organ unique to schizasterids (Ziegler, 2014). Here, we provide the first characterization of the schizasterid IC from macroscopic to ultrastructural levels based on observations of dozens of specimens of *B. townsendi*. These novel observations reveal a number of unique structural features of the organ.

The internal anatomy of both schizasterid species described in the present study is highly similar, and akin to their previously studied congeners *B. latifrons* and *A. cavernosus* (Ziegler, 2014). At the histological level, most of the digestive tract of *B. townsendi* resembles that of other heart urchins (Koehler, 1883; Hamann, 1887; Anisimov, 1982; De Ridder and Jangoux, 1993; Holland, 2013). Despite the absence of haemal ducts in both intestine and IC, our observations suggest that the small haemal space within the wall of the IC is continuous with the enlarged haemal space of the intestine via the tissues comprising the caecal canal and mesentery suspending the organ (Figures 1f–i, 2d), thus permitting an exchange of haemal fluid between intestinal and IC walls.

At the ultrastructural level, the intestinal tissues of *B. townsendi* resemble those of the distantly related heart urchin *Echinocardium cordatum* (De Ridder and Jangoux, 1993). As in *E. cordatum*, the intestinal ECM of *B. townsendi* is characterized by the presence of electron-dense granules (Figure 3a). These particles have previously been identified as a ferric phosphate precipitate caused by oxidative deposition under reducing conditions (Buchanan et al., 1980). In addition, the digestive epithelium of the intestine in both spatangoid species is characterized by the presence of a dense cover of microvilli. In contrast, the IC’s enterocytes possess only a small number of tiny microvilli (Figure 3c). This sparse cover of microvilli in the IC is likely related to the dense bacterial population attached to the organ’s digestive epithelium (Figure 3d). A further difference between intestinal and IC walls is the strong basal folding of the IC’s enterocytes (Figure 3b), indicative of an increased exchange between these cells and the underlying ECM (De Ridder and Jangoux, 1993). Furthermore, the presence of a pseudo-stratified coelomic epithelium in the intestine (Figure 3a) as opposed to a mono-layered coelomic epithelium in the IC (Figure 3b) suggests the possibility that these two tissues experience different mechanical loads. However, arguably the most unusual structural feature of the IC is the fact that the organ is lined by podocytes on its exterior, coelomic side (Figure 3h). The presence of this cell type is usually indicative of ultrafiltration mediated by a muscle-generated pressure differential (Ruppert, 1994). However, in comparison to the intestine, the IC features only a small number of myoepithelial cells. If exchange does indeed occur across this barrier, it could be caused by pressure generated via the contraction of myoepithelial cells elsewhere in the digestive or mesenterial systems, or through diffusion.

In most marine deposit feeders, the main contents of the digestive tract are sediment particles. This is true for *B. townsendi* as well, as previously shown for its congener *B. fragilis* (Eichelbaum, 1910). However, the IC again is strikingly different from the rest of the digestive tract in this respect: although it is directly connected to the sediment-packed intestine by the caecal canal, we observed no sediment particles inside the ICs of *B. townsendi* and other schizasterids. Instead, the organ was filled with an organic mass composed of diverse microorganisms (Figure 2g).

### Composition of the Microbiome of the Schizasterid Intestinal Caecum

An increasing number of studies are beginning to shed light on the gut microbiomes found in various echinoderm taxa, among them several deposit-feeding sea cucumber (Gao et al., 2014; Yamazaki et al., 2016; Wang et al., 2018; Pagán-Jiménez et al., 2019; Weigel, 2020) and sea urchin species (Thorsen, 1999; Thorsen et al., 2003; da Silva et al., 2006; Schwob et al., 2020). However, most of these analyses focus on microbial organisms found in the main digestive tract, while the present study provides data permitting a comparison between the microbiomes found inside the main gut compartments and a specialized digestive tract derivative.

Our genomic analyses show that the IC of *B. townsendi* contains a community of predominantly anaerobic bacteria with Bacteroidetes averaging 34.6% of the microbiome, in addition to Desulfobacterales (16.0%) and Spirochaetales (9.2%). Similar results were obtained for *A. cordatus*, with Desulfobacterales averaging 31.5% of its microbiome, followed by Bacteroidetes (14.0%) and Spirochaetales (9.5%), with the addition of Rhizobiales (9.5%) and Lentisphaerales (7.0%). The most common phylotypes in *A. cordatus* were closely related to a recently described genus of the Desulfobacterales thus far known from a single psychrophilic species, *Desulfoconvexum algidum*, isolated from a fjord in the Svalbard archipelago (Konneke et al., 2013) and members of the bacterial candidate phylum Gracilibacteria, which recent genomic analyses suggest are often symbiotic, displaying limited metabolic capacity (Sieber et al., 2019).

For *B. townsendi*, the IC microbiome was found to be significantly different in composition and diversity from that of the rest of the gut (Figure 5A). Members of the sulfate-reducing Desulfobacteriaceae were very abundant in the IC, in contrast to the intestine and rectum, where members of the Desulfobulbaceae and Desulfuromonadaceae were prominent (Supplementary Table S1). Desulfobacteriaceae are commonly found in marine sediments and are known to fully oxidize fermentative decomposition products such as acetate (Dhillon et al., 2003; Leloup et al., 2007; Jackson et al., 2014; Na et al., 2015). Another sulfate-reducing lineage abundant in the IC libraries were members of the genus *Desulfocarbo*, a taxon within Desulfarculales recently discovered from a coal bed in central North America (An and Picardal, 2014). OTUs closely related to another uncultured group of Deltaproteobacteria, FW113, whose metabolic function is unknown, were also abundant. This group has also been found in anaerobic marine sediments and microbial mats (Liang et al., 2006; Harris et al., 2013).

Confirming our microscopic results, OTUs most closely related to spirochetes were also very abundant in the ICs of *B. townsendi* and *A. cordatus*, but were not recovered in any of the other gut compartments. In contrast, a recent study found spirochetes primarily of the genus *Spirochaeta* in abundance in the main gut tissue of the schizasterid *Abatus agassizii*, and identified these taxa as part of its gut microbiome (Schwob et al., 2020). However, this particular species of *Abatus* does not possess an IC (Ziegler, 2014). In fact, the spirochete lineages found in the ICs of *B. townsendi* and *A. cordatus* differ significantly from those in *A. agassizii*, being predominantly related to the cultured taxon *Sediminispirochaeta* and the uncultured *Spirochaeta* group 2 clade (Supplementary Tables S1, S2). *Sediminispirochaeta* is a recently reclassified genus (Shivani et al., 2016) composed of three type strains isolated from microbial mats and oil fields (Fracek and Stolz, 1985; Magot et al., 1997; Dubinina et al., 2015). Spirochetes are known to form symbiotic associations with diverse metazoan taxa including cnidarians, arthropods, mollusks, echinoderms, and mammals (Paster and Canale-Parola, 1982; Margulis and Hinkle, 2006; Lawler et al., 2016; Van de Water et al., 2016; Jackson et al., 2018). Perhaps the best-studied example is the termite hindgut symbiosis, which includes free-living spirochetes in the gut fluids as well as spirochetes living on and in symbiotic flagellated protists (Smith and Arnott, 1974; Leadbetter et al., 1999; Iida et al., 2000; Graber and Breznak, 2004; Dröge et al., 2006, 2008; Brune and Dietrich, 2015). This scenario is similar to what was here observed in the schizasterid IC, where some spiral-shaped bacteria were attached to the IC’s digestive epithelium (Figure 4h) and others were found free-floating in the lumen of the organ (Figure 2g). In termites, spirochetes are known to facilitate the digestion of lignocellulose, providing metabolizable, small molecules to the host (Breznak and Brune, 1994). However, pure culture studies have shown that these spirochetes also participate in CO_2_-reducing acetogenesis (Leadbetter et al., 1999), thus providing a different pathway of carbon delivery to the host. Both endo- and ectosymbiotic members of the Bacteroidetes have additionally been identified in these termite gut associations (Wenzel et al., 2003; Noda et al., 2005). In the present study, members of the latter bacterial taxon were also found in the IC. For example, relatives of the genus *Draconibacterium*, previously cultured from marine sediments in East Asia (Du et al., 2014; Gwak et al., 2015), were abundant in the organ. However, the most prominent OTU inside the IC was related to the uncultured VC2.1 Bac22 group, first described from a hydrothermal vent on the Mid-Atlantic Ridge (Reysenbach et al., 2000), but since found to be common in marine habitats as well as in association with various metazoan taxa such as annelids and cetaceans (Alain et al., 2002; Bik et al., 2016).

Finally, relatively low numbers of an OTU closely related to *Sulfurimonas*, a sulfur-oxidizing lineage of the Thiovulaceae (Epsilonproteobacteria), were observed in eight of the twelve (66.7%) IC samples from *B. townsendi*, but were particularly abundant in one sample (Supplementary Table S1). In addition to oxidizing reduced sulfur compounds, which would be in abundance in the IC due to the presence of sulfate-reducing bacteria, members of this group are known for reducing nitrate (Han and Perner, 2015). However, these organisms require at least a partially oxic environment for their metabolism. While we did not measure oxygen levels in this study, low concentrations of O_2_ have previously been detected in the IC of *B. latifrons* (Plante and Jumars, 1992).

The strong and consistent distinction between the microbial communities found in the IC vs. other gut compartments (Figure 5B) suggests selection for specific microbial taxa in the IC. While the exact mechanisms of colonization or selection in the IC are yet to be determined, the bacteria in many animal-microbe symbioses are initially acquired from the environment (Ruby and Lee, 1998; Dubilier et al., 2008). In spatangoids, diverse microorganisms are constantly ingested through the sediment and seawater diet of the host (De Ridder and Foret, 2001). As the initial microbial community passes through the gut, the transient microbiome may change due to the interactions of microbes with gut contents or their host as well as the conditions inside the digestive tract. Recently, such a selection process was inferred to occur in the digestive tract of the schizasterid *A. agassizii* (Schwob et al., 2020). However, in marine deposit feeders that lack hindgut diverticula, most of this modified microbial community is likely washed out once the sediment reaches the anus. In turn, the IC found in *B. townsendi* is a discrete organ separate from the main flow of sediment, so that its inhabitants are protected from washout. We hypothesize that once the IC forms during ontogeny, it becomes initially seeded with bacteria from the host’s diet, and then its microbial community rapidly diverges in composition from that of the rest of the gut. Future studies using naïve animals would be important to address this topic, but conditions suitable for laboratory cultivation of *B. townsendi* have not been achieved thus far.

### Function of the Microbiome of the Schizasterid Intestinal Caecum

The IC microbiome of *B. townsendi* is both consistent among individuals (although not completely uniform, suggesting some host or environmental variability) and strikingly different in composition and diversity from other gut compartments. These characteristics suggest that it has a distinct role in its host’s biology. However, some possible functions of the IC microbiome may be ruled out already on the basis of its position along the digestive tract, i.e., at the posterior end of the intestine, just anterior to the relatively short rectum. For example, it seems improbable that the IC is of importance in detoxifying gut contents, which are about to be shed from the body as feces via the anus. The organ also is unlikely to be involved in stockpiling beneficial microbes that could be used to inoculate the rest of the gut, for example to protect against the development of communities of pathogenic microbes or to facilitate digestive processes in other gut compartments.

Instead, the position of the IC is more consistent with a role in nutrition. It has previously been argued that conditions in the hindgut of marine deposit feeders are likely beneficial for bacteria (Plante et al., 1990), and that microbial activity in the posterior digestive tract should come at no or little cost to the host. If activities of such a hindgut microbial community produce molecules of use to the host that can be taken up across the gut wall, then selection might lead to the evolution of intestinal pouches to increase surface area for product uptake. Incidentally, the residence time of the microbial communities in such diverticula can be high, allowing them to become distinct from the microbiomes in gut compartments with only a short residence time. All of our data on the position, structure, and microbiome of the IC are consistent with this general scenario.

What products of use to the host might then be generated by the schizasterid IC’s microbiome? We saw no evidence for phagocytosis of microbes by enterocytes of the organ, so it seems unlikely that *B. townsendi* is gardening bacteria for direct consumption. Instead, we hypothesize that the schizasterid IC’s microbiome converts dissolved organic compounds from the intestinal fluid into smaller, more easily metabolizable molecules that can then be used by the host. Intestinal fluid may be moved into the IC via pressure generated by muscular contraction in other gut compartments (for example, by peristalsis, which has been observed in spatangoids: De Ridder et al., 1985), by cilia near the entrance of the IC, or by diffusion. Dissolved organic molecules in the inbound liquid may fuel microbial activities inside the IC, much as occurs in the termite hindgut (Leadbetter et al., 1999). Products of these activities might be returned to the host in several ways. Small molecules like fatty acids could diffuse across the IC wall into the somatocoel or they might be taken up by IC enterocytes through pinocytosis and then transported into the haemal space of the organ. Once inside the haemal lacunae, such products could then be circulated directly to other parts of the body or be ultrafiltered from the IC’s haemal space across the podocytes lining the exterior of the IC.

A better understanding of these processes requires a detailed understanding of the organic substrates provided to the IC, and especially of the metabolic interactions of the diverse microbes in this specific gut compartment. At this point we can only speculate about these interactions. Although autotrophic members of the Desulfobacteriaceae and Spirochaetales are known, it is more likely that they are functioning heterotrophically in the present context, as most described species of these groups as well as the Bacteroidetes are chemoorganotrophic carbon degraders and are most likely involved in fermentation of complex carbon substrates. The ensuing simpler molecules such as short-chained fatty acids might then serve as carbon source to the sulfate-reducing bacteria. This is reminiscent of a recently described association between a marine sediment spirochete and a sulfate-reducing *Desulfovibrio* strain that were studied in co-culture, and where the spirochete produces fermentation products for the sulfate reducer (Shivani et al., 2016). However, in echinoderms active sulfate reduction can lead to toxicity for the host in these types of symbioses (De Ridder and Foret, 2001; De Ridder and Saucède, 2020; Schwob et al., 2020), but this may be ameliorated by sulfur-oxidizing bacteria observed in the IC that remove sulfide from the organ. Tests of these hypotheses will require a comprehensive functional characterization of the IC’s microbial community.

The present data show that each schizasterid IC contains a complex microbial consortium composed of taxa with overlapping and complementary metabolic activities. In this regard, the organ resembles another spatangoid hindgut structure, the recto-intestinal caecum (RIC), which is present in a distantly related group of heart urchins (De Ridder, 1994; De Ridder and Saucède, 2020). Morphologically and structurally, this organ differs from the IC (Ziegler, 2014) and in *E. cordatum* typically contains several small nodules predominantly composed of Deltaproteobacteria, Bacteroidetes, and Firmicutes (61.5, 23.1, and 15.4%, respectively; da Silva et al., 2006). However, the sulfur-oxidizing bacteria found inside the RIC were shown to be filamentous bacteria that form layers surrounding a detrital particle covered with sulfate-reducing microbes (De Ridder and Brigmon, 2003). The bacteria on the outside of these nodules oxidize thiosulfate and sulfide originating from its core, thus providing the host with acetate and propionate (Temara et al., 1991, 1993; Brigmon and De Ridder, 1998; Thorsen, 1998). In total, the processes inside the RIC contribute about 10% to the energy budget of the animal (Thorsen, 1999; Thorsen et al., 2003). The oxygen required to support part of this symbiosis is supplied through the somatocoel (Thorsen, 1999). Though neither the nodules nor the specific microbial taxa involved in this symbiosis are present in the schizasterid IC, similarly complex metabolic interactions are likely to have evolved among IC microbial residents. Whether the ciliates sometimes observed in the organ also play a role in these processes remains unclear at present.

### Evolutionary and Ecological Considerations

Previous work has shown that the IC is absent in most extant higher schizasterid taxa as well as in more distantly related spatangoids (Ziegler, 2014). Thus, it seems probable that the IC evolved within a subclade of the Schizasteridae. However, until now, taxon sampling was limited, making inferences on the evolutionary history of the IC difficult.

**FIGURE 8:**
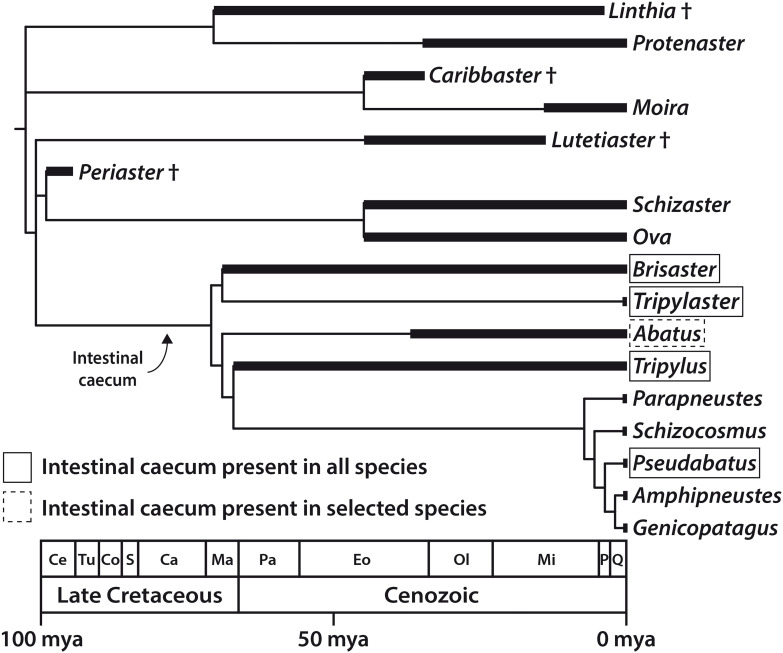
Distribution of the intestinal caecum (IC) in the Schizasteridae and inferred timing of the origin of the IC and, by inference, of its microbiome; evolutionary tree of the Schizasteridae constructed by calibrating a cladogram against the known fossil record. Morphology-based phylogeny modified from Stockley et al. (2005) and Stara et al. (2016), stratigraphic data taken from Lambert (1933), Clark (1937), McKinney et al. (1988), Markov (1994), Lindley (2001), and Kroh and Smith (2010). Ca, Campanian; Ce, Cenomanian; Co, Coniacian; Eo, Eocene; Ma, Maastrichtian; Mi, Miocene; mya, million years ago; Ol, Oligocene; P, Pliocene; Pa, Paleocene; Q, Quaternary; S, Santonian; Tu, Turonian.

With the addition of observations reported in this study, a clearer picture emerges (Figure 8). The IC is present in all species of *Brisaster*, *Tripylaster*, *Tripylus*, and *Pseudabatus*-note that *P. nimrodi* has previously been placed in the genus *Abatus*, but is here tentatively re-assigned to *Pseudabatus* based on two recent phylogenetic analyses (Van Oosterom, 2012; Stara et al., 2016). However, the presence of the IC varies in the genus *Abatus*: the organ has been observed in the two species analyzed here (Table 1), but it is absent in four other species (Ziegler, 2014). In addition, the absence of an IC is here confirmed for the species *Parapneustes cordatus*, which in a previous study had been assigned to *Tripylus* (Ziegler, 2014). Furthermore, the IC is not present in any of the other extant schizasterid genera analyzed so far (Ziegler, 2014). An examination of the distribution of the IC in a phylogenetic context suggests that this structure may have arisen only once, in the common ancestor of a group of at least nine schizasterid genera including *Brisaster*, *Tripylus*, and *Pseudabatus* (Figure 8). If this hypothesis is correct, then the IC has been lost on several occasions: at least once within the genus *Abatus*, and more than once in the clade comprising *Parapneustes* and *Genicopatagus* as well as other genera (Figure 8). An alternative hypothesis, of course, is that the IC has originated more than once.

We note that there is variation in the form and relative size of the IC among the schizasterid species that possess the organ. While in most species, including *B. townsendi* and *A. cordatus*, it is a slightly curved cylinder constituting about 0.5–1.5% of TV, it is bean-shaped and shorter in *A. cavernosus* (Ziegler, 2014), more rectangular in *T. excavatus*, and reduced in *P. nimrodi*. This variation in shape and size may be due to diversification after a single origin, or due to more than one independent origin of the IC. Though the latter scenario does not seem particularly parsimonious given the strong similarity in overall shape and location of the IC, it is worth noting that barriers to the evolution of digestive appendices may be low (Smith et al., 2017). Distinguishing among the different evolutionary hypotheses will require both an improved understanding of schizasterid phylogeny (David et al., 2005, 2016; Saucède et al., 2013), as well as further study of the distribution and characteristics of the IC within the clade.

Although the systematics of schizasterids are not yet fully resolved, the available data nonetheless allow us to infer the minimum age of the IC. Spatangoids are a relatively ancient sea urchin lineage that originated in the Mesozoic, more precisely the Early Cretaceous, approximately 135 mya (Kroh and Smith, 2010). About 40 million years later, the first schizasterids (members of the fossil genus *Periaster*) appeared during the early phase of the Late Cretaceous (Figure 8). However, the two oldest schizasterid taxa known to possess the IC, i.e., *Brisaster* and *Tripylus*, originated in the final phase of the Late Cretaceous, about 70–66 mya (Forbes, 1845; Lambert, 1933; Markov, 1994; Kroh and Smith, 2010). It is therefore very likely that at least in these two genera, the IC (and by inference, it’s microbiome) evolved prior to the Cretaceous-Paleogene asteroid impact that occurred about 65.5 mya. This cataclysmic event caused a significant reduction in metazoan diversity both in marine and terrestrial habitats (Renne et al., 2013), although various marine taxa, including spatangoids, apparently were not as strongly affected by the ensuing mass extinction (Smith and Jeffery, 1998; Ribeiro et al., 2011; Lowery et al., 2018). A better understanding of the physiological benefit of the IC’s digestive symbiosis might help to elucidate whether the presence of the organ in selected schizasterid taxa constituted an evolutionary advantage in a post-extinction ocean with reduced oxygen and nutrient levels (Henehan et al., 2019).

While schizasterids represent a relatively old spatangoid lineage, they nonetheless share with other heart urchins a specific infaunal lifestyle (Nichols, 1959; Gibbs, 1963; Bromley and Asgaard, 1975; Kanazawa, 1992). Correspondingly, ecological data show that schizasterids are frequently found in close association with other spatangoid taxa (McCauley and Carey, 1967; Lie and Kisker, 1970; Nichols, 1975, 2003; Thompson et al., 1987, 1993; Allen et al., 1999; Stull et al., 2001; Widdicombe et al., 2004; Macdonald et al., 2012). However, the physical and chemical parameters of the surrounding sediment may change significantly over short distances and slight depths, leading to zonation effects that provide heart urchins with potentially narrow ecological niches (Gibbs, 1963; Bromley et al., 1995; Enderlein, 2004). For example, in contrast to spatangoid taxa such as *Echinocardium*, *Brissus*, or *Brissopsis* that bury down to 10–20 cm depth (Kanazawa, 1992), species of *Brisaster* have been reported to be shallow burrowers that only dig down to 1–5 cm below the sediment surface (Gibbs, 1963; Brownell, 1970; Kanazawa, 1992). These observations could indicate that the schizasterid IC can only be found in species that inhabit a specific zone below the sediment surface where conditions of the surrounding sediment are suitable to support the IC’s digestive symbiosis. In fact, the reduction or even complete absence of the organ in schizasterids such as *Pseudabatus, Amphipneustes*, or *Brachysternaster* as well as most species of *Abatus* (Ziegler, 2014) could be related to a change in lifestyle, as these taxa have been observed to be secondarily semi-infaunal or even entirely epifaunal organisms (Larrain, 1985; Pearse and McClintock, 1990; David et al., 2005; Thompson and Riddle, 2005; Sumida et al., 2008; Schwob et al., 2020). A test for this hypothesized change in lifestyle would be a comparison of denuded tests of the different schizasterid species, as shape of the anterior ambulacrum correlates with burrowing depth and sediment structure (Mortensen, 1951; Chesher, 1963; Kanazawa, 1992; Bromley et al., 1995). In addition to providing a more comprehensive picture of the role of the IC and its microbiome, such data might aid our understanding of the notable evolutionary success of schizasterids in the Southern Ocean (Poulin et al., 2002; Schwob et al., 2020).

## Conclusion

Our data suggest that the microbial inhabitants of the IC play a distinct, if not yet fully understood, nutritional role in the biology of their hosts, and in at least some schizasterid lineages have done so since the Mesozoic. Understanding exactly how the schizasterid IC’s microbiome interacts with the host organism will require further study of the constituent microorganisms and of the integrated holobiont. Functional metagenomics, cultivation, and physiological investigations of selected microbial lineages, paired with tracer studies of material transport from the IC to the host, are likely to be particularly effective approaches with which to address these open questions.

## Data Availability Statement

Raw and derived image data are available from the MorphoBank online repository (Ziegler et al., 2020). These data include two 3D MRI scans, two conventional and four contrast-enhanced 3D μCT scans, as well as 37 media files amounting to a total data volume of 2.7 GB. Bacterial 16S rRNA gene sequences were submitted to the Sequence Read Archive at the National Center for Biotechnology Information and have been assigned biosample accession numbers SAMN14692096 to SAMN14692121 under project number PRJNA627958.

## Ethics Statement

Because the study of echinoderms and most other invertebrates is not regulated by the Animal Welfare Act (United States), approval for this study by the California State University Long Beach Institutional Animal Care and Use Committee was not required. Collections were conducted under Scientific Collecting Permit SC-7296 (California Department of Fish and Wildlife, Sacramento, CA, United States).

## Author Contributions

AZ and BP conceived the study. AZ, AG, JD, and BP carried out the experiments, analyzed the data, and wrote the manuscript. All authors contributed to the article and approved the submitted version.

## Conflict of Interest

The authors declare that the research was conducted in the absence of any commercial or financial relationships that could be construed as a potential conflict of interest.
