# Supplementary material for: Schizasterid Heart Urchins Host Microorganisms in a Digestive Symbiosis of Mesozoic Origin
Source: Front Microbiol. 2020 Jul 22;11:1697. doi: 10.3389/fmicb.2020.01697 (PMC7387435; doi:10.3389/fmicb.2020.01697)
Supplement: Supplementary file 1 [file Table_1.docx]

Table S1. Abundance and identification of operational taxonomic units (OTUs) with at least 2,000 sequences among *Brisaster townsendi* digestive tract samples.

| **OTU** | **Phylum** | **Lowest**  **taxonomic**  **level** | **Stomach**  **(N=2)** | | **Intestine**  **(N=2)** | | **Intestinal caecum**  **(N=12)** | | | | | | | | | | | | **Rectum**  **(N=2)** | |
| --- | --- | --- | --- | --- | --- | --- | --- | --- | --- | --- | --- | --- | --- | --- | --- | --- | --- | --- | --- | --- |
| 1 | Unassigned | Unassigned | 1691 | 475 | 0 | 0 | 0 | 0 | 0 | 0 | 0 | 0 | 0 | 0 | 0 | 0 | 0 | 0 | 0 | 0 |
| 2 | Bacteroidetes | Uncult. BD2-2 grp. | 0 | 0 | 1385 | 1099 | 0 | 0 | 0 | 0 | 0 | 0 | 0 | 0 | 0 | 0 | 0 | 0 | 1091 | 1190 |
| 3 | Bacteroidetes | Chitinophagaceae | 1361 | 693 | 0 | 0 | 0 | 0 | 0 | 0 | 0 | 0 | 0 | 0 | 0 | 0 | 0 | 0 | 6 | 0 |
| 4 | Bacteroidetes | *Lutibacter* | 0 | 0 | 667 | 642 | 0 | 0 | 0 | 0 | 0 | 0 | 0 | 0 | 0 | 0 | 0 | 0 | 853 | 759 |
| 5 | Bacteroidetes | *Lutibacter* | 0 | 0 | 847 | 438 | 0 | 0 | 3 | 0 | 0 | 0 | 0 | 0 | 0 | 0 | 0 | 0 | 911 | 1099 |
| 6 | Deltaproteobacteria | Uncult. Sva0081 grp. | 36 | 0 | 590 | 524 | 0 | 0 | 0 | 0 | 0 | 0 | 0 | 0 | 0 | 0 | 0 | 0 | 610 | 580 |
| 7 | Deltaproteobacteria | Desulfobulbaceae | 0 | 0 | 649 | 569 | 0 | 0 | 0 | 0 | 0 | 0 | 0 | 0 | 0 | 0 | 0 | 0 | 447 | 519 |
| 8 | Deltaproteobacteria | Desulfobulbaceae | 0 | 0 | 2946 | 2724 | 0 | 0 | 0 | 0 | 0 | 0 | 0 | 0 | 0 | 0 | 0 | 0 | 1988 | 2637 |
| 9 | Deltaproteobacteria | Uncult. Sva1033 grp. | 0 | 0 | 1334 | 1192 | 0 | 0 | 0 | 0 | 0 | 0 | 0 | 0 | 0 | 0 | 0 | 0 | 1091 | 1372 |
| 10 | Deltaproteobacteria | Sandaracinaceae | 25 | 7 | 1904 | 1231 | 0 | 0 | 0 | 0 | 0 | 0 | 0 | 0 | 0 | 0 | 0 | 0 | 1239 | 1185 |
| 11 | Deltaproteobacteria | Uncult. Sva0485 grp. | 0 | 0 | 639 | 574 | 0 | 0 | 0 | 3 | 0 | 0 | 0 | 0 | 0 | 0 | 0 | 0 | 477 | 570 |
| 12 | Deltaproteobacteria | Syntrophobacteraceae | 24 | 36 | 1804 | 1407 | 0 | 0 | 0 | 0 | 0 | 0 | 0 | 0 | 0 | 0 | 0 | 0 | 1353 | 1387 |
| 13 | Gammaproteobacteria | Uncult. B2M28 grp. | 95 | 194 | 601 | 497 | 0 | 0 | 0 | 0 | 0 | 0 | 0 | 0 | 0 | 0 | 0 | 0 | 487 | 496 |
| 14 | Gammaproteobacteria | Uncult. B2M28 grp. | 73 | 62 | 655 | 553 | 0 | 0 | 0 | 0 | 0 | 0 | 0 | 0 | 0 | 0 | 0 | 0 | 576 | 524 |
| 15 | Gammaproteobacteria | *Ralstonia* | 1250 | 1099 | 28 | 9 | 0 | 0 | 5 | 0 | 0 | 0 | 0 | 0 | 0 | 0 | 0 | 0 | 0 | 0 |
| 16 | Alphaproteobacteria | *Mesorhizobium* | 374 | 1011 | 194 | 101 | 12 | 38 | 47 | 27 | 18 | 66 | 12 | 14 | 47 | 33 | 16 | 10 | 238 | 170 |
| 17 | Bacteroidetes | *Draconibacterium* | 17 | 14 | 0 | 0 | 0 | 17279 | 0 | 3826 | 2860 | 0 | 3069 | 245 | 21917 | 2552 | 2916 | 4526 | 61 | 40 |
| 18 | Bacteroidetes | *Draconibacterium* | 0 | 0 | 0 | 0 | 0 | 1697 | 0 | 0 | 188 | 0 | 0 | 0 | 0 | 0 | 0 | 248 | 0 | 0 |
| 19 | Bacteroidetes | Uncult. VC2.1 Bacc22 grp. | 1500 | 191 | 79 | 23 | 21785 | 8882 | 2 | 32122 | 25932 | 6 | 26719 | 24566 | 7 | 20004 | 22699 | 18419 | 759 | 629 |
| 20 | Epsilonproteobacteria | *Sulfurimonas* | 0 | 0 | 0 | 0 | 10 | 14 | 9 | 8 | 0 | 3162 | 0 | 8 | 33 | 190 | 0 | 0 | 0 | 0 |
| 21 | Patescibacteria | Uncult. JGI 0000069-P22 grp. | 6 | 0 | 0 | 0 | 0 | 718 | 0 | 289 | 0 | 0 | 317 | 10 | 670 | 260 | 262 | 0 | 0 | 0 |
| 22 | Patescibacteria | Gracilibacteria | 0 | 19 | 0 | 0 | 784 | 0 | 0 | 538 | 0 | 0 | 617 | 0 | 765 | 1037 | 253 | 0 | 0 | 0 |
| 23 | Deltaproteobacteria | *Desulfocarbo* | 0 | 21 | 0 | 0 | 42 | 876 | 468 | 146 | 443 | 0 | 314 | 121 | 0 | 0 | 13 | 226 | 5 | 9 |
| 24 | Deltaproteobacteria | *Desulfocarbo* | 0 | 0 | 0 | 0 | 0 | 146 | 1148 | 1247 | 36 | 0 | 20 | 0 | 250 | 28 | 42 | 78 | 0 | 0 |
| 25 | Deltaproteobacteria | *Desulfocarbo* | 0 | 21 | 0 | 0 | 186 | 850 | 1050 | 537 | 159 | 0 | 312 | 345 | 675 | 986 | 242 | 159 | 0 | 0 |
| 26 | Deltaproteobacteria | *Desulfocarbo* | 0 | 0 | 0 | 0 | 0 | 0 | 1399 | 0 | 0 | 1361 | 32 | 0 | 0 | 0 | 41 | 0 | 0 | 0 |
| 27 | Deltaproteobacteria | Desulfobacteraceae | 0 | 0 | 0 | 0 | 231 | 179 | 1666 | 228 | 376 | 1290 | 126 | 12 | 94 | 1024 | 61 | 899 | 0 | 0 |
| 28 | Deltaproteobacteria | Desulfobacteraceae | 0 | 0 | 0 | 0 | 55 | 50 | 1497 | 288 | 72 | 0 | 145 | 17 | 32 | 223 | 47 | 423 | 0 | 14 |
| 29 | Deltaproteobacteria | Desulfobacteraceae | 0 | 0 | 0 | 0 | 215 | 65 | 766 | 204 | 119 | 0 | 169 | 25 | 106 | 103 | 125 | 262 | 0 | 0 |
| 30 | Deltaproteobacteria | *Desulfovibrio* | 10 | 0 | 0 | 0 | 380 | 285 | 6255 | 347 | 132 | 110 | 249 | 37 | 98 | 204 | 65 | 201 | 0 | 0 |
| 31 | Deltaproteobacteria | Uncult. FW113 grp. | 0 | 0 | 0 | 0 | 174 | 128 | 1997 | 263 | 104 | 0 | 100 | 63 | 85 | 0 | 88 | 123 | 0 | 0 |
| 32 | Deltaproteobacteria | Uncult. FW113 grp. | 0 | 0 | 0 | 0 | 0 | 989 | 941 | 143 | 169 | 0 | 228 | 159 | 610 | 0 | 0 | 54 | 0 | 0 |
| 33 | Deltaproteobacteria | Uncult. FW113 grp. | 36 | 0 | 0 | 0 | 206 | 127 | 0 | 251 | 211 | 0 | 244 | 228 | 286 | 138 | 268 | 147 | 0 | 0 |
| 34 | Deltaproteobacteria | Uncult. FW113 grp. | 0 | 0 | 0 | 0 | 36 | 0 | 0 | 402 | 323 | 0 | 410 | 105 | 224 | 211 | 127 | 219 | 21 | 0 |
| 35 | Deltaproteobacteria | Uncult. FW113 grp. | 0 | 0 | 0 | 0 | 148 | 801 | 4735 | 539 | 594 | 0 | 323 | 53 | 698 | 531 | 311 | 1089 | 0 | 15 |
| **OTU** | **Phylum** | **Lowest**  **taxonomic**  **level** | **Stomach**  **(N=2)** | | **Intestine**  **(N=2)** | | **Intestinal caecum**  **(N=12)** | | | | | | | | | | | | **Rectum**  **(N=2)** | |
| 36 | Deltaproteobacteria | Uncult. FW113 grp. | 0 | 0 | 0 | 0 | 0 | 0 | 225 | 1446 | 145 | 0 | 119 | 0 | 248 | 792 | 204 | 547 | 0 | 0 |
| 37 | Deltaproteobacteria | Uncult. FW113 grp. | 0 | 0 | 0 | 0 | 0 | 0 | 676 | 65 | 70 | 15115 | 27 | 59 | 309 | 1559 | 163 | 103 | 0 | 0 |
| 38 | Deltaproteobacteria | Uncult. FW113 grp. | 0 | 0 | 0 | 0 | 83 | 260 | 1626 | 299 | 82 | 0 | 90 | 45 | 395 | 452 | 61 | 66 | 0 | 0 |
| 39 | Deltaproteobacteria | Uncult. PB19 grp. | 7 | 0 | 0 | 0 | 142 | 203 | 363 | 409 | 232 | 0 | 193 | 90 | 44 | 144 | 294 | 385 | 0 | 0 |
| 40 | Deltaproteobacteria | Uncult. PB19 grp. | 0 | 0 | 0 | 0 | 0 | 0 | 9006 | 186 | 136 | 250 | 162 | 0 | 386 | 0 | 0 | 283 | 0 | 0 |
| 41 | Spirochaetes | *Sediminispirochaeta* | 12 | 0 | 0 | 0 | 74 | 937 | 2212 | 756 | 151 | 0 | 375 | 20 | 0 | 287 | 524 | 351 | 0 | 4 |
| 42 | Spirochaetes | *Sediminispirochaeta* | 0 | 0 | 0 | 0 | 112 | 166 | 937 | 162 | 52 | 1474 | 129 | 76 | 379 | 176 | 98 | 234 | 0 | 0 |
| 43 | Spirochaetes | Uncult. *Spirochaeta*  2 grp. | 0 | 0 | 0 | 0 | 269 | 382 | 2466 | 439 | 413 | 263 | 317 | 148 | 289 | 338 | 224 | 536 | 0 | 0 |
| 44 | Spirochaetes | Uncult. *Spirochaeta*  2 grp. | 0 | 0 | 0 | 0 | 0 | 7 | 1649 | 0 | 0 | 1640 | 0 | 0 | 26 | 0 | 0 | 8 | 0 | 0 |
| 45 | Spirochaetes | Uncult. *Spirochaeta*  2 grp. | 29 | 14 | 0 | 0 | 1071 | 1840 | 8543 | 2255 | 852 | 7615 | 788 | 969 | 3340 | 1503 | 1083 | 2212 | 0 | 0 |
