# Supplementary material for: Schizasterid Heart Urchins Host Microorganisms in a Digestive Symbiosis of Mesozoic Origin
Source: Front Microbiol. 2020 Jul 22;11:1697. doi: 10.3389/fmicb.2020.01697 (PMC7387435; doi:10.3389/fmicb.2020.01697)
Supplement: Supplementary file 2 [file Table_2.docx]

Table S2. Abundance and identification of operational taxonomic units (OTUs) with at least 150 sequences among *Abatus cordatus* ICs.

| **OTU** | **Phylum** | **Lowest**  **taxonomic**  **level** | **Intestinal caecum**  **(N=4)** | | | |
| --- | --- | --- | --- | --- | --- | --- |
| 1 | Nanoarchaeota | Woesearchaeia | 3509 | 390 | 843 | 112 |
| 2 | Unknown bacterial phylum | Unassigned | 0 | 0 | 0 | 241 |
| 3 | Unknown bacterial phylum | Unassigned | 0 | 250 | 0 | 0 |
| 4 | Unknown bacterial phylum | Unassigned | 13 | 0 | 0 | 265 |
| 5 | Acidobacteria | Thermoanaerobaculaceae | 0 | 892 | 0 | 0 |
| 6 | Bacteroidetes | Bacteroidales | 0 | 375 | 0 | 0 |
| 7 | Bacteroidetes | Uncult. BD2-2 grp. | 0 | 0 | 2856 | 270 |
| 8 | Bacteroidetes | Uncult. BD2-2 grp. | 0 | 0 | 0 | 404 |
| 9 | Bacteroidetes | Uncult. BD2-2 grp. | 15 | 38 | 13 | 93 |
| 10 | Bacteroidetes | *Carboxylicivirga* | 0 | 338 | 36 | 0 |
| 11 | Bacteroidetes | *Carboxylicivirga* | 0 | 99 | 271 | 73 |
| 12 | Bacteroidetes | *Draconibacterium* | 0 | 315 | 0 | 0 |
| 13 | Bacteroidetes | Uncult. Bac22 grp. | 109 | 56 | 154 | 97 |
| 14 | Bacteroidetes | Uncult. Bac22 grp. | 409 | 0 | 0 | 0 |
| 15 | Bacteroidetes | Bacteroidia | 168 | 6 | 14 | 0 |
| 16 | Bacteroidetes | Chitinophagaceae | 0 | 0 | 0 | 1648 |
| 17 | BRC1 | Uncult. BRC1 grp. | 0 | 122 | 149 | 22 |
| 18 | Cyanobacteria | Sericytochromatia | 0 | 0 | 15 | 343 |
| 19 | Kiritimatiellaeota | Uncult. MSBL3 grp. | 414 | 9 | 19 | 8 |
| 20 | Kiritimatiellaeota | Uncult. MSBL3 grp. | 5554 | 1742 | 35 | 44 |
| 21 | Kiritimatiellaeota | Uncult. WCHB1-41 grp. | 95 | 16 | 15 | 2375 |
| 22 | Lentisphaerae | Uncult. PRD18C08 grp. | 0 | 52 | 3238 | 316 |
| 23 | Lentisphaerae | Uncult. *P.palm*C41 grp. | 530 | 172 | 702 | 0 |
| 24 | Lentisphaerae | Uncult. *P.palm*C41 grp. | 0 | 8 | 472 | 24 |
| 25 | Lentisphaerae | Uncult. *P.palm*C41 grp. | 203 | 39 | 24 | 8129 |
| 26 | Marinimicrobia | Uncult. AB16 grp. | 118 | 1456 | 0 | 12 |
| 27 | Patescibacteria | Gracilibacteria | 0 | 0 | 5238 | 277 |
| 28 | Patescibacteria | Uncult. JGI 0000069-P22 grp. | 0 | 251 | 0 | 0 |
| 29 | Patescibacteria | Gracilibacteria | 14518 | 2489 | 556 | 1267 |
| 30 | Planctomycetes | Uncult. *Urania*-1B-19 grp. | 0 | 320 | 240 | 0 |
| 31 | Unclassified Proteobacteria | Unassigned | 24 | 12 | 0 | 1039 |
| 32 | Alphaproteobacteria | *Rhizobium leguminosarum* | 0 | 356 | 0 | 21 |
| 33 | Alphaproteobacteria | *Mesorhizobium* | 565 | 0 | 0 | 0 |
| 34 | Alphaproteobacteria | Xanthobacteraceae | 211 | 0 | 0 | 0 |
| 35 | Deltaproteobacteria | Desulfobacteraceae | 115 | 157 | 49 | 75 |
| 36 | Deltaproteobacteria | *Desulfoconvexum* | 2371 | 8 | 893 | 199 |
| 37 | Deltaproteobacteria | *Desulfoconvexum* | 102 | 125 | 31 | 75 |
| 38 | Deltaproteobacteria | *Desulfoconvexum* | 204 | 379 | 0 | 0 |
| 39 | Deltaproteobacteria | *Desulfoconvexum* | 751 | 233 | 847 | 150 |
| 40 | Deltaproteobacteria | *Desulfoconvexum* | 2814 | 7906 | 40 | 198 |
| 41 | Deltaproteobacteria | *Desulfoconvexum* | 574 | 3132 | 8423 | 1227 |
| 42 | Deltaproteobacteria | Uncult. FW113 grp. | 0 | 1357 | 0 | 6 |
| 43 | Deltaproteobacteria | Uncult. Oligoflexales 0319-6G20 grp. | 0 | 2216 | 0 | 0 |
| 44 | Deltaproteobacteria | Uncult. PB19 grp. | 115 | 44 | 26 | 61 |
| 45 | Deltaproteobacteria | Uncult. PB19 grp. | 9 | 0 | 0 | 487 |
| 46 | Deltaproteobacteria | Uncult. SAR324 grp. | 0 | 175 | 0 | 0 |
| 47 | Epsilonproteobacteria | *Sulfurospirillum* | 0 | 1899 | 5173 | 1549 |
| 48 | Epsilonproteobacteria | *Sulfurimonas* | 0 | 95 | 0 | 179 |
| 49 | Gammaproteobacteria | *Ralstonia* | 0 | 47 | 134 | 1761 |
| 50 | Spirochaetes | *Sediminispirochaeta* | 405 | 0 | 0 | 0 |
| 51 | Spirochaetes | *Sediminispirochaeta* | 0 | 220 | 0 | 0 |
| 52 | Spirochaetes | Uncult. *Spirochaeta* 2 grp. | 0 | 0 | 182 | 45 |
| 53 | Spirochaetes | Uncult. *Spirochaeta* 2 grp. | 0 | 618 | 0 | 20 |
| **OTU** | **Phylum** | **Lowest**  **taxonomic**  **level** | **Intestinal caecum**  **(N=4)** | | | |
| 54 | Spirochaetes | Uncult. *Spirochaeta* 2 grp. | 0 | 105 | 307 | 0 |
| 55 | Spirochaetes | Uncult. *Spirochaeta* 2 grp. | 0 | 0 | 0 | 469 |
| 56 | Spirochaetes | Uncult. *Spirochaeta* 2 grp. | 87 | 133 | 27 | 21 |
| 57 | Spirochaetes | Uncult. *Spirochaeta* 2 grp. | 1624 | 688 | 4977 | 483 |
| 58 | Spirochaetes | Uncult. *Spirochaeta* 2 grp. | 0 | 0 | 0 | 548 |
